# Intratumoral heterogeneity and clonal evolution in liver cancer

**DOI:** 10.1038/s41467-019-14050-z

**Published:** 2020-01-15

**Authors:** Bojan Losic, Amanda J. Craig, Carlos Villacorta-Martin, Sebastiao N. Martins-Filho, Nicholas Akers, Xintong Chen, Mehmet E. Ahsen, Johann von Felden, Ismail Labgaa, Delia DʹAvola, Kimaada Allette, Sergio A. Lira, Glaucia C. Furtado, Teresa Garcia-Lezana, Paula Restrepo, Ashley Stueck, Stephen C. Ward, Maria I. Fiel, Spiros P. Hiotis, Ganesh Gunasekaran, Daniela Sia, Eric E. Schadt, Robert Sebra, Myron Schwartz, Josep M. Llovet, Swan Thung, Gustavo Stolovitzky, Augusto Villanueva

**Affiliations:** 1 Department of Genetics and Genomic Sciences, Cancer Immunology Program, Tisch Cancer Institute, Icahn School of Medicine at Mount Sinai, New York, NY USA; 2 Icahn Institute for Data Science and Genomic Technology, Icahn School of Medicine at Mount Sinai, New York, NY USA; 3 Diabetes, Obesity and Metabolism Institute, Icahn School of Medicine at Mount Sinai, New York, NY USA; 4 Division of Liver Diseases, Department of Medicine, Liver Cancer Program, Tisch Cancer Institute, Graduate School of Biomedical Sciences, Icahn School of Medicine at Mount Sinai, New York, NY USA; 5 Department of Pathology, University of Sao Paulo School of Medicine, Sao Paulo, Brazil; 6 Adaptive Biotechnologies, Seattle, WA USA; 7 I. Department of Medicine, University Medical Center Hamburg-Eppendorf, Hamburg, Germany; 8 Department of Visceral Surgery, Lausanne University Hospital CHUV, Lausanne, Switzerland; 9 Liver Unit and Centro de Investigación Biomédica en Red de Enfermedades Hepáticas y Digestivas (CIBERehd), Clínica Universidad de Navarra, Pamplona, Spain; 10 Immunology Institute, Icahn School of Medicine at Mount Sinai, New York, NY USA; 11 Department of Pathology, Dalhousie University, Halifax, NS Canada; 12 Department of Pathology, Icahn School of Medicine at Mount Sinai, New York, NY USA; 13 Department of Surgery, Icahn School of Medicine at Mount Sinai, New York, NY USA; 14 Sema4, a Mount Sinai venture, Stamford, CT USA; 15 Liver Cancer Translational Research Laboratory, BCLC Group, IDIBAPS, Hospital Clinic, Universitat de Barcelona, Barcelona, Catalonia Spain; 16 Institució Catalana de Recerca i Estudis Avançats, Barcelona, Catalonia Spain; 17 IBM T. J. Watson Research Center, Yorktown Heights, New York, NY USA; 18 Division of Hematology and Medical Oncology, Department of Medicine, Icahn School of Medicine at Mount Sinai, New York, NY USA

**Keywords:** Cancer, Immunology, Gastroenterology, Oncology

## Abstract

Clonal evolution of a tumor ecosystem depends on different selection pressures that are principally immune and treatment mediated. We integrate RNA-seq, DNA sequencing, TCR-seq and SNP array data across multiple regions of liver cancer specimens to map spatio-temporal interactions between cancer and immune cells. We investigate how these interactions reflect intra-tumor heterogeneity (ITH) by correlating regional neo-epitope and viral antigen burden with the regional adaptive immune response. Regional expression of passenger mutations dominantly recruits adaptive responses as opposed to hepatitis B virus and cancer-testis antigens. We detect different clonal expansion of the adaptive immune system in distant regions of the same tumor. An ITH-based gene signature improves single-biopsy patient survival predictions and an expression survey of 38,553 single cells across 7 regions of 2 patients further reveals heterogeneity in liver cancer. These data quantify transcriptomic ITH and how the different components of the HCC ecosystem interact during cancer evolution.

## Abstract (web summary)

Immune-mediated selection pressures impact the clonal evolution of tumours. Here, in hepatocellular carcinoma the authors map spatio-temporal interactions between tumor and immune cells, highlighting the regulatory substrate of intra-tumoural heterogeneity that correlates with regional adaptive immune responses.

## Introduction

Primary liver cancer is the fourth cause of cancer-related mortality worldwide. With more than 750,000 new cases annually (33,000 in the United States (US)), it has become the fastest growing malignancy in the United States (US), both in terms of incidence and mortality^1^. Hepatocellular carcinoma (HCC) is the most frequent form of liver cancer and it generally develops in the context of chronic liver disease due to viral hepatitis B or C, alcohol abuse and non-alcoholic fatty liver disease. Hepatitis B virus (HBV) infection is the main cause of HCC worldwide, and the World Health Organization estimates that 257 million people are living with HBV. Despite the clinical efficacy of molecular therapies in HCC patients at advanced stages^1^, the almost inevitable emergence of drug resistance stands in the way of a definitive cure. The ability of cancer cells to adapt to pharmacological pressures can be described in terms of tumor evolution, and stems from the intrinsic diversity, or heterogeneity of cancer^2^. Cancer heterogeneity defines the distinct genetic alterations and phenotypes between cancer cells within the same tumor nodule (i.e., intratumor heterogeneity or ITH) or between different tumor nodules within the same patient. ITH can have major clinical consequences, as falsely classifying subclonal mutations as clonal drivers may misdirect treatment decisions. This sampling bias can potentially impact decision-making when using molecular information derived from a single tissue biopsy, as recently described in lung cancer^3^.

Multiregional tumor sampling has helped to characterize ITH, both at the phenotypic and genetic levels, in an attempt to reconstruct phylogenetic and spatio-temporal relationships of geographically distant tumor regions^4^. An emergent theme from these studies is that the spatio-temporal dynamics of ITH are not entirely captured by DNA somatic mutations alone^3^. Even though tumors are complex ecosystems incorporating nontumoral cells, few studies have addressed how the tumor microenvironment, in particular the immune system, contributes to ITH. A recent study integrated DNA sequencing data, gene expression, and T-cell clonality from multiple tumor sites of ovarian tumors to report heterogeneous cancer-immune interactions highly suggestive of immunoediting^5^. Along these lines, another study found different activation states of the immune system during the transition from in situ to invasive breast cancer^6^, with intriguing evidence of co-evolution of cancer and immune cells. These reports underscore the importance of understanding the interactions between cancer and immune cells within tumor ecosystems, especially considering the remarkable success of immune checkpoint inhibitors in heterogeneous solid tumors^7^. In this context, HCC offers a unique opportunity to determine the contribution of tumor and viral antigens in immune activation, a feature relatively unexplored using immunogenomics.

Results from two phase 2 clinical trials using PD-1 inhibitors suggest that a subgroup of HCC patients (~18%) significantly benefit from immune checkpoint inhibition^1^. We hypothesize that a better understanding of the interactions between HCC and the immune system can help identify biomarkers of response to these therapies. To investigate the natural history of these interactions, we integrated data (i.e., RNA-seq, DNA targeted sequencing, TCR sequencing, and DNA copy number changes) from multiple regions of 14 HCC resection specimens, including single-cell RNA-seq data from seven regions of two patients. Leveraging our unique multiregional dataset, we used an immunogenomics approach to find evidence of a tumor-driven adaptive immune response correlating with ITH. Our model suggests that tumor neoantigens dominantly recruit tumor infiltrating lymphocytes (TILs) compared to other sources of antigens (e.g., HBV, cancer testis antigens (CTAs)). Furthermore, we uncover strong regional differences in transcriptional factor networks at the single-cell level.

## Results

### Regional clonal immune responses fuel ITH in HCC

**Fig. 1:**
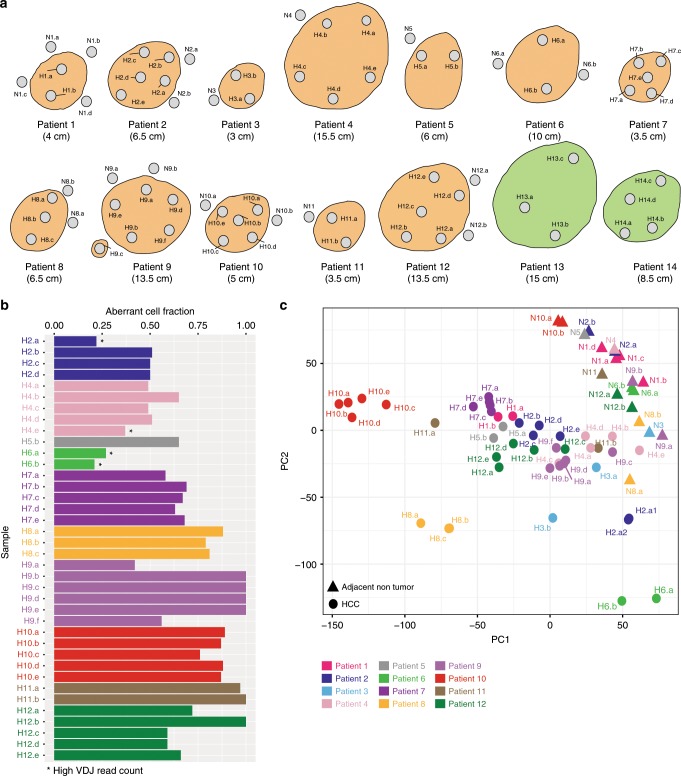
Summary of sampling and tumor purity data. **a** Geographic distribution of the multiregional sampling (H: HCC sample; N: Nontumoral adjacent; Orange: Samples bulk sequenced; Green: Samples single-cell sequenced). **b** Regional tumor cell purity determined with ASCAT. **c** PCA of tumor and nontumor regions of all patients included in RNA-seq (Circles: HCC; Triangles: Adjacent nontumor).

We compiled a dataset of 71 multiregional samples from 14 HCC patients, including 51 tumoral and 20 nontumoral adjacent regions (median of 3.5 tumor and 1.5 nontumor regions per patient, including a technical replicate for region A of patient 2 (P02)). All patients except P09 had single-nodule early stage HCC (Barcelona Clinic Liver Cancer stage A)^8^ and were treated with surgical resection without any prior therapy (Fig. 1a). Most patients were male (64%, 9/14), with a median age of 63 years, and a median tumor size of 65 mm. As expected, considering that the underlying liver disease was predominantly due to HBV (50%, 7/14), the majority of patients did not have severe fibrosis in the adjacent nontumoral liver (63% (7/11) as quantified using the METAVIR score^9^ (Supplementary Table 1). Histological evaluation of tumor grade and immune infiltrate demonstrated phenotypic ITH in 50% (7/14) of patients (Supplementary Fig. 1a, b). Regional variations in tumor purity were confirmed using DNA data from genotyping arrays. The regions with the lowest tumor cell purity as determined with ASCAT (version 2.4)^10^ were the ones with the highest immune infiltrate on histological examination (Fig. 1b).

To assess regional transcriptomic heterogeneity, we evaluated major axes of variability of the gene expression data using multidimensional scaling (MDS). As expected, there was a clear separation between tumor and nontumor regions and furthermore, for most patients, all tumor regions tended to colocalize (Fig. 1c). There were 5/12 (40%) patients (P02, P03, P04, P09, and P11) with at least one region closer to those of another patient than to the other regions of the same tumor. When we integrated data of TIL burden, we found that most patients with outlier regions had heterogeneous distribution of immune infiltrate as per histological evaluation (P02, P03, P09, and P11). This suggested that tumor-immune infiltrate could be a major determinant of transcriptomic ITH and motivated us to study the regional interactions between cancer and immune cells using immunogenomics.

**Fig. 2:**
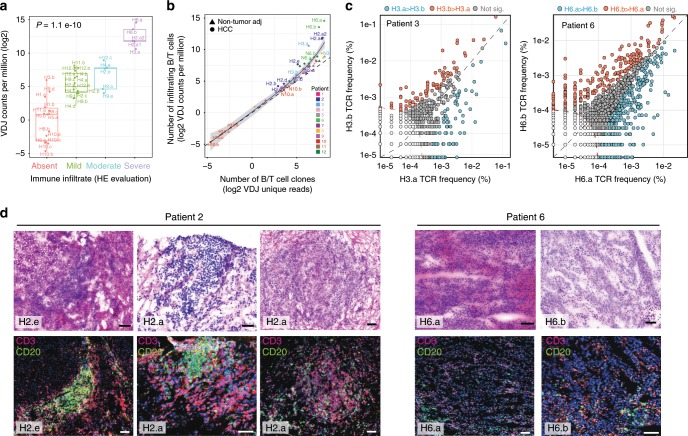
Immunogenomic view of regional cancer-immune interactions in HCC and immune infiltrates. **a** Number of RNA-seq reads mapping to VDJ loci grouped by pathological immune infiltrate assessment (ANOVA, error bars = SE, *N* = 38 samples. For each boxplot, the centre line represents the median. Upper and lower limits of each box represent the 75th and 25th percentiles, respectively. The whiskers represent the lowest data point still within 1.5× box size of the lower quartile and the highest data point still within 1.5× box size of the upper quartile). **b** Number of RNA-seq reads mapping to VDJ as a function of the number of unique reassembled CDR3 sequences (i.e., number of unique immune clones). **c** Scatter plot of TCR rearrangement frequencies between tumoral regions of patient 3 and 6. TCR rearrangements found at significantly higher frequencies in region H3.a/H6.a than H3.b/H6.b are filled in blue. TCR rearrangements found at significantly higher frequencies in region H3.b/H6.b than H3.a/H6.a are filled in red. **d** Paired H&E and immunofluorescence of CD3 and CD20 in high TIL burden regions of P02 and P06 (CD3: Red; CD20: Green; black bar 100 µm; white bar 50 µm, *N* = 3 independent experiments). Source data are provided as a Source Data file.

To better characterize the intensity and characteristics of regional TIL burden in HCC, we first quantified the B and T cell receptor (B/TCR) RNA-seq reads mapping to VDJ loci and normalized by total library size in all samples. The nontargeted and sparse nature of these data prevents a deep characterization of the TIL receptor repertoire. Nevertheless, previous studies confirmed the validity of RNA-seq data to infer immune clonotypes and to provide a reasonable proxy of TCR diversity in tumor samples^11^. We found that tumor regions classified as having severe immune infiltrate on histology had significantly more RNA-seq reads mapping to the VDJ loci than those classified as having less immune infiltrate (*P* = 1.1e−10) (Fig. 2a). We confirmed significant ITH in TIL burden in P02, P03, and P06 with higher VDJ read count in regions H2.a and H2.e compared to H2.b, H2.c, and H2.d, in region H3.a compared to H3.b, and in H6.a compared to H6.b (Fig. 2b). These estimates were confirmed with TCR sequencing for patients 3 and 6. This is consistent with a recent study that reported ITH in HCC immune infiltrate^12^.

We next sought to quantify the degree of T-cell clonality in the different regions of the same tumor. We hypothesized that differences in TIL burden across regions could be due to differences in local immune clonal expansions. We conducted TCR sequencing (ImmunoSeq) in multiple regions of P03 and P06. Despite the fact that we did not find significant differences in overall T cell clonality between the different regions of P03 and P06, there were significant differences in the number of unique T-cell expansions detected in the different regions of these patients (Fig. 2c). Regions H3.a and H6.a had significantly more unique T-cell expansions than H3.b and H6.b, respectively. To better understand the nature of the regional differences in TIL, we examined the architecture of the TILs in P02, P03, and P06 using immunofluorescence for T (CD3) and B (CD20) cell markers. We detected tertiary lymphoid structures (TLS, confirmed with PNAd staining for High-Endothelial Venules (Supplementary Fig. 2a)) in some of the regions of P02 and P03, but not in P06, where T cells had a diffuse distribution intermingled with cancer cells (Fig. 2d). TLS are transient ectopic lymphoid organizations that develop in nonlymphoid tissues functioning as important sites for the initiation and/or maintenance of immune responses. In HCC, the presence of intratumoral TLS correlates with a lower risk of tumor recurrence^13^. Interestingly, P02 had regional differences in the distribution of immune cells with both TLS and diffuse pattern in H2.a, whereas H2.e predominantly had TLS alone. This, combined with our previous findings of low TIL burden in H2.e compared to H2.a, prompted us to hypothesize that TLS density is a proxy for the timing of the interaction between cancer and immune cells. To test this, we used (1) quantification of relative fractions of immune cell subsets using data deconvolution methods^14^, and (2) T-cell cytotoxicity as measured by the Immune Cytolytic Activity Index^15^. Compared to H2.e, region H2.a had a higher proportion of memory B-cells, CD8-T cells, CD4 memory cells, and macrophages, suggestive of a more mature immune response (Supplementary Fig. 2b). Additionally, T-cell cytotoxicity was higher in H2.a compared to H2.e (Supplementary Fig. 2c). These data prompted us to further examine the regional interface between cancer and immune cells and its contribution to transcriptomic ITH.

### Predicted neoepitope immunogenicity is spatially variable

To study the interactions between cancer and immune cells, we first computed the predicted immunogenicity of tumor neoepitopes across the different regions of all tumors. Despite these predictions being suboptimal compared to directly identifying presented epitopes via mass spectroscopy^16^, there is evidence suggesting that in silico predicted binding affinities form useful priors for immunologic reactivity^17^. First, we called expressed somatic mutations using RNA-seq data. Despite being inferior to DNA mutation calling, numerous reports demonstrate the usefulness of RNA-seq-based mutation calling^18^. There was significant heterogeneity in the distribution of expressed somatic mutations across regions, with an average per patient ranging from 70 (P06) to 225 (P03) (Supplementary Fig. 3a, Supplementary Data File 1). However, we found a clear patient-specific clustering of somatic mutations (Supplementary Fig. 3b). To determine if this regional heterogeneity in expressed mutations was also affecting known HCC drivers, we conducted targeted DNA deep sequencing of the 58 genes most frequently mutated in HCC. We confirmed a clonal distribution of known drivers, such as *TERT* promoter, *CTNNB1*, and *TP53* (Supplementary Fig. 3c, Supplementary Table 2), with only one tumor region (H4.a) depicting a subclonal mutation of *CTNNB1*^19^. Using DNA Sanger sequencing, we validated 11 expressed mutations predicted as damaging (Supplementary Table 3).

We then estimated in silico regional differences in neoepitope distribution by assessing putative immunogenicity of the expressed mutations. We first allelotyped the samples for all six HLA class I molecules. HLA-I alleles were stably expressed across all regions except for P05 (Supplementary Fig. 4, Supplementary Data File 2). We combined the expressed HLA-I alleles and mutations (i.e., predicted neoepitopes) using the well-established netMHC algorithm^20^ to estimate the binding affinity of each HLA/neo-peptide combination across tumor regions. This binding affinity quantifies the likelihood of a given neoepitope being presented on the surface of a tumor cell and potentially being recognized by a T cell. While a critical binding affinity of a neoepitope to a given HLA-I allele is required for actual immunogenicity, it is only suggestive compared to directly identifying presented epitopes via mass spectroscopy^16^. However, mounting evidence suggests in silico predicted binding affinities are useful for vaccine design and disease diagnostics^21^.

**Fig. 3:**
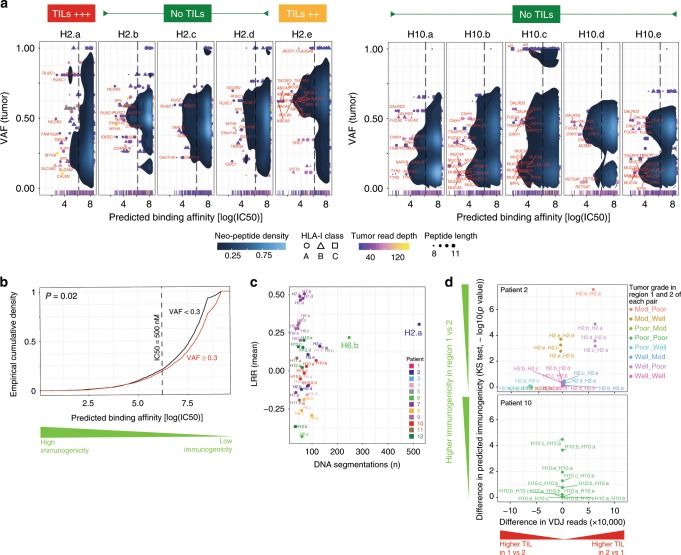
Neoantigen binding affinity. **a** 2D density of log-scaled peptide binding affinity as a function of the VAF of somatic mutations across regions of P10 and P02. Dotted line depicts 50% inhibitory concentration (IC50) = 500 nM (lower IC50 means stronger binding and higher immunogenicity, HLA-I class A: circle; HLA-I class B: Triangle; HLA-I class C: square). **b** Empirical cumulative density plot of log-scaled binding affinity distribution for neoantigens according to VAF of expressed mutations. Kolmogorov-Smirnov test with one-sided alternative hypothesis. p-value is for rejecting the null in favor of the alternative. **c** Log R Ratio (LRR) mean as a function of DNA segmentation for each tumoral region. **d** *Y*-axis depicts one-sided Kolmogorov-Smirnov test p-value for regional sample pairs of neoantigen binding affinity profiles, i.e., a quantification of the relative shift of putative immunogenicity between paired tumor regions. *X*-axis depicts the difference in the number of RNA-seq reads mapped to the VDJ locus between the first and second region of each pair, i.e., the regional differences in adaptive immune burden. Sample pairs are colored based on tumor grade. Source data are provided as a Source Data file.

We predicted between 41 and 466 HLA/neoepitope combinations per tumor region (Supplementary Data File 2). We conducted multiregional comparison of the distribution of predicted neoepitope binding affinities. This overcomes the difficulty of comparing distinct HLA alleles from different patients using data from single biopsies. In P02, H2.e is significantly more immunogenic than H2.a (*P* < 1e−8), followed by regions H2.d, H2.c, and H2.b (*P* = 2e−4, *P* = 5e−4, *P* = 1e−3, respectively, Fig. 3a). A similar heterogeneous pattern holds in P09, P04, P03, and P05 (Supplementary Fig. 5). In contrast, all regions of P10 are predicted to be similarly immunogenic, consistent with those of P08, P06, P01, P11, and P12 (Fig. 3a, Supplementary Fig. 5). Expressed clonal mutations, as defined by a variant allele fraction (VAF) higher than 0.3 tend to emit fewer immunogenic neoepitopes than subclonal mutations (Fig. 3b). This was confirmed using whole-exome sequencing (WES) data in P10. In all the regions analyzed in this patient, the predicted immunogenicity of subclonal mutations was significantly higher than for clonal mutations (Supplementary Fig. 6). Defining passenger mutations by their expression in only some regions of the same tumor nodule, i.e. branch mutations, we also observed a significant increase in predicted immunogenicity compared to mutations present in all regions (trunk mutations), (*P* = .02). These included the candidate HCC driver genes *TP53*, *CTNNB1*, and *NFE2L2* identified in our targeted DNA sequencing panel. Indeed, all three driver mutations give rise to neoepitope distributions with average binding affinity greater than 1000 nM. The threshold of 500 nM is used to define high binding affinity and select peptides as candidates for cancer vaccines^22^. This is consistent with the expectation that early somatic driver mutations should be immune-evasive.

In the patient with the highest TIL heterogeneity (P02), we found an association between changes in regional neoepitope immunogenicity and TIL burden. Region H2.e has the most putatively immunogenic neoepitopes while H2.a has the least, and yet H2.a has the greatest TIL burden in that patient. Given that tumor grade in H2.a is poorly differentiated, followed by H2.e (i.e., moderately differentiated) and the other regions (i.e., well differentiated), it is suggestive that the adaptive immune response in H2.a has edited the tumor to be much less immunogenic, termed negative selection. P02, along with P06 (highest TIL burden overall), both had regions with significantly higher DNA segmentations as shown by CNV analysis (Fig. 3c). This reflects intrinsic genetic differences in tumor cells in these regions which, in addition to TIL burden, likely contribute to the transcriptomic heterogeneity we find in our RNA-seq data (Fig. 1c). Conversely, in the patient with the lowest TIL burden, P10, we find minor variations among the regional neoepitope predicted immunogenicity (Fig. 3d). In addition, we find that key immune checkpoint genes (e.g., *CTLA4, PDCD1, CD274*) are upregulated and correlated with TIL burden, indicative of an inhibitory response toward T-cell activation (Supplementary Fig. 7).

### Limited contribution of HBV and CTAs to TIL recruitment

**Fig. 4:**
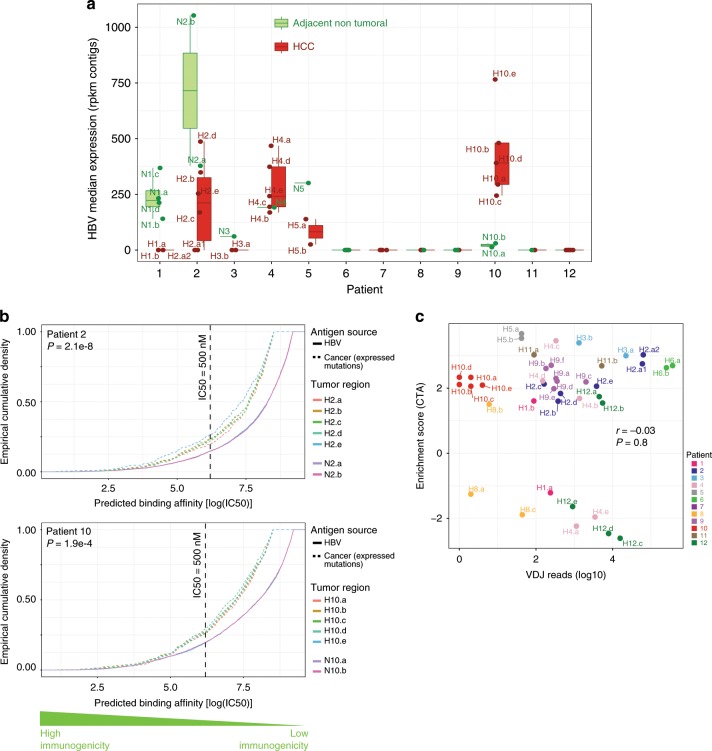
HBV antigen binding affinity and CTA immunogenicity. **a** Expression distribution of HBV for adjacent nontumoral and tumoral regions across patients (HCC: Red; Adjacent nontumoral: Green, error bars = SE, *N* = 44 samples. For each boxplot, the centre line represents the median. Upper and lower limits of each box represent the 75th and 25th percentiles, respectively. The whiskers represent the lowest data point still within 1.5× box size of the lower quartile and the highest data point still within 1.5× box size of the upper quartile). **b** Empirical cumulative density plot of log-scaled binding affinity distribution across regions for both HBV and tumor neoepitopes. Kolmogorov-Smirnov test with one-sided alternative hypothesis. p-value is for rejecting the null in favor of the alternative. **c** Correlation plot of CTA enrichment score and RNA-seq reads mapped to the VDJ locus across regions. Source data are provided as a Source Data file.

In patients with HBV-related HCC, the relative contribution of tumor neoantigens and HBV antigens to TIL recruitment is unknown. In principle, TILs should respond to any nonself peptides, regardless of their tumoral or viral origin. To elucidate the role of HBV in TIL recruitment, we first evaluated expression of HBV transcripts assembled from RNA-seq reads not mapping to the human genome, and ultimately used them to predict immunogenicity of HBV antigens. These antigens can arise from HBV covalently closed circular DNA or HBV insertions in DNA malignant hepatocytes. We found patients with strong variability in HBV expression between tumor and adjacent nontumoral tissue (Fig. 4a), a feature previously reported in HBV-related HCC^23^. Furthermore, we observed variation in HBV expression in different tumoral regions of patients P02, P04, and P10, including some regions showing no expression of HBV transcripts (e.g., region H2.a of P02). This suggests differential selection pressure on infected tumor clones. We also found evidence of HBV DNA integrations, including the previously described *FN1* (Supplementary Table 4) integration. Importantly, when compared to tumoral neoepitopes from any given region, the predicted binding affinity of HBV peptides is shifted towards lower binding affinity than mutation-derived neoepitopes (*P* = 2.1e−8, *P* = 1.9e−4 for P02 and P10, respectively; Fig. 4b). This suggests that in HBV driven HCC tumors, neoepitopes dominate HBV epitopes in their recruitment of TILs. Though such a suggestion awaits confirmation via mass spectroscopy, we also note the reported impact of HCC-cell differentiation in HBV replication^24^, which could further decrease the pool of HBV epitopes competing with tumor neoantigens.

Next, we evaluated self-antigens as another source for TIL recruitment, as they are known constituents of the cancer antigenome^25^. CTAs are among the better-studied tumor antigens^26^, they are frequently re-expressed in HCC, and they have been evaluated as candidate cancer vaccines. We did not compute their putative immunogenicity because they are self-proteins. When considering gene expression of the whole gene family^26^, Gene Set Enrichment Analysis of CTAs showed a heterogeneous enrichment across our multiregional dataset, mainly for patients P01, P04, P09, and P12. However, CTA enrichment score was not correlated with TIL infiltrate either by histological assessment or VDJ read count. As in previous reports, our data suggest that CTA expression alone seems insufficient to elicit an intratumoral immune response^27^ (Fig. 4c).

### ITH gene expression signatures predict outcomes in HCC

**Fig. 5:**
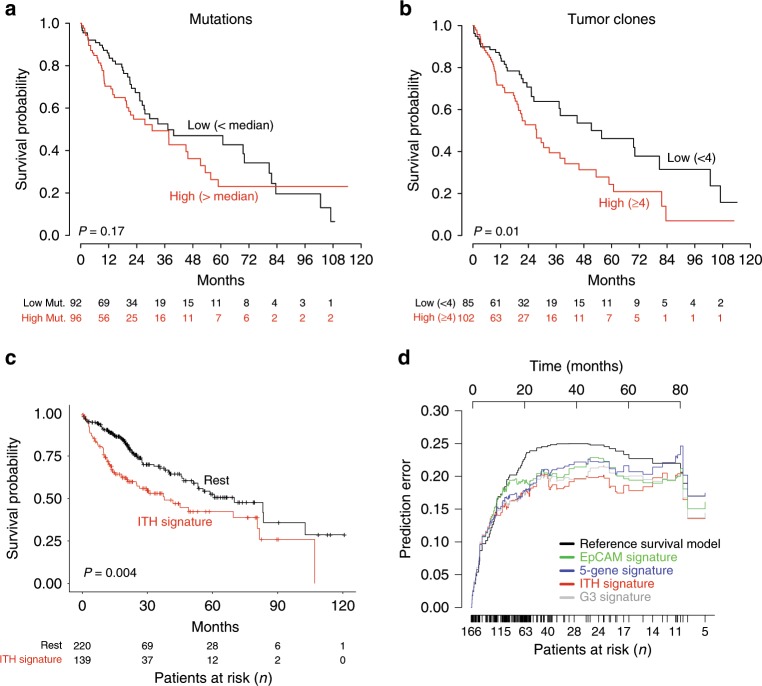
Survival analyses of ITH models in TCGA-HCC. **a** Kaplan-Meier curves with HCC DNA mutations and **b** DNA-based clonality estimates. **c** Kaplan-Meier curve for overall survival in the TCGA dataset after patients are classified based on the ITH signature. **d** Prediction error curves of competing parametric Cox proportional hazard models depicting time dependent Brier score for models built from principal components of the G3, EpCAM, 5-gene, and ITH signatures. Survival analysis was done using the Kaplan-Meier (log-rank) test.

Given the high scale of ITH we observed from the immune, neoantigen, and HBV antigen analysis enabled by multiregional sampling, we next sought to characterize the gene expression landscape of ITH. We hypothesized that intratumoral differential gene expression would capture important clonal and immune evolution information in HCC. If true, this signal should have survival impact in a cohort of single-biopsy HCC. To test this, and also directly address their clinical relevance, we used our multiregional gene expression dataset for feature selection and leveraged the TCGA-HCC Cohort^28^ as a testing set for the survival correlations. We first assessed known readouts of tumor clonality in the TCGA-HCC Cohort by calling DNA-based somatic mutations for each tumor within the TCGA-HCC Cohort with whole-exome sequencing data (WES) available (*N* = 188). DNA-based tumor clonality outperformed number of DNA mutations to predict survival in TCGA-HCC, suggesting that DNA mutation number might be a suboptimal proxy of ITH in HCC (Fig. 5a, b) and underscoring the possible role of ITH in survival prediction.

We performed all possible pairwise regional differential expression comparisons between tumor regions in patients with at least three regions sampled. These comparisons can be interpreted as principal spatial axes for tumor gene expression, highlighting the rich dynamics that underlie ITH. Using nested-cross-validation (see Methods, Supplementary Fig. 8a, b) to simultaneously control for overfitting and hyperparameter adjustment, we iteratively learned optimal combinations of regional differential expression signatures and computed the Integrated Brier Score on holdout test-sets to evaluate their prognostic power on the TCGA-HCC dataset. The IBS score measures the goodness of prediction for censored data, which essentially quantifies the accuracy of prognostic predictions in survival analysis with Cox regression^29^. The set of genes differentially expressed between region H2.a and regions H2.b-c-d-e of patient 2 had the lowest Integrated Brier Score (i.e., prediction error) for survival in the TCGA-HCC dataset. We used a procedure of variance ranking^30^ to select the minimum number of genes required to retain the predictive power of the P02 gene set. The resulting ITH signature (363 genes, Supplementary Data File 3) was detected in 38% (139/359) of patients in the TCGA-HCC dataset and it was associated with significantly worse survival (*N* = 359, Fig. 5c). Reasoning that our ITH signature from P02 rivals current single-biopsy prognostic HCC signatures (e.g., G3, 5-gene or EpCAM signatures^31^), we compared their prognostic accuracy by comparing their Integrated Brier Score as a function of survival time (Fig. 5d). The ITH signature has the lowest prediction error compared to any of the best single-biopsy based predictors and the least optimistic discrimination index across an additional repeated cross-validation analysis (*P* < 0.03, Supplementary Fig. 8c). The ITH signature was also correlated with early tumor recurrence in the Heptromic Cohort (Supplementary Fig. 8d), as well as with higher levels of the poor prognostic biomarker alpha-fetoprotein (Supplementary Fig. 8d). In this dataset, the performance of the ITH signature was similar to the other single-biopsy prognostic signatures (Supplementary Fig. 8e).

### Single-cell RNA-seq reveals regulatory ITH

**Fig. 6:**
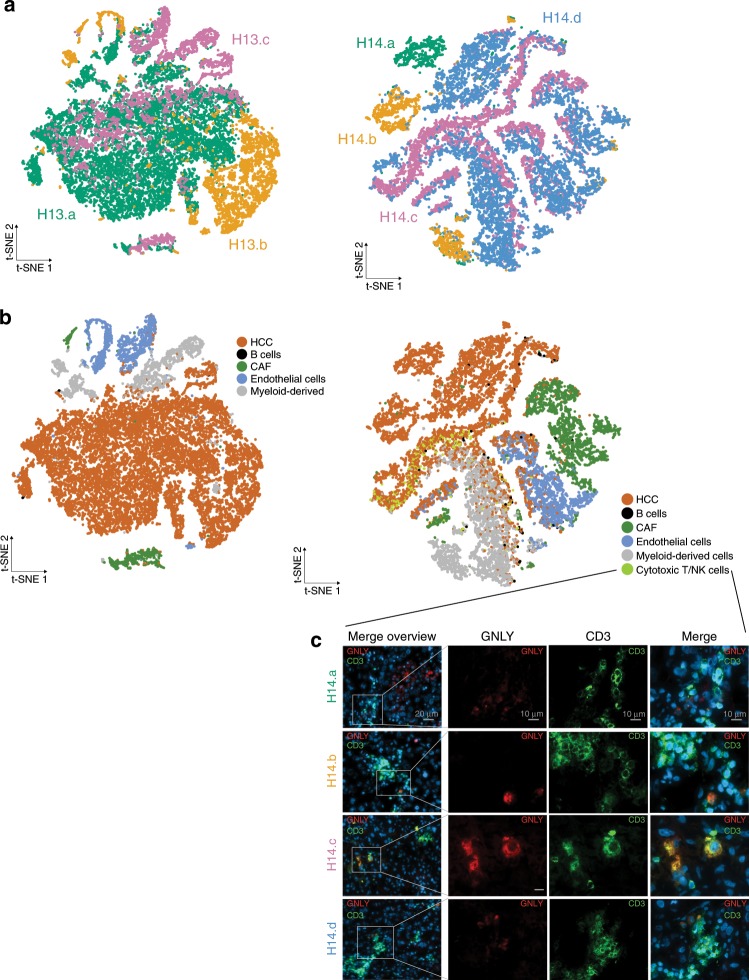
HCC ecosystem and regional transcriptomic heterogeneity on single-cell RNA-seq. **a** t-SNE plots of single-cell clusters colored by tumor region (H13.a: Green; H13.b: Yellow; H13.c: Pink) and **b** affiliation to cell lineage by gene expression. **c** Immunofluorescence staining for GNLY (red) and CD3 (green) in P14. Scale bar is 20 μm long in merge overview panels and 10 μm for all other panels. *N* = 3 independent experiments. Source data are provided as a Source Data file.

**Fig. 7:**
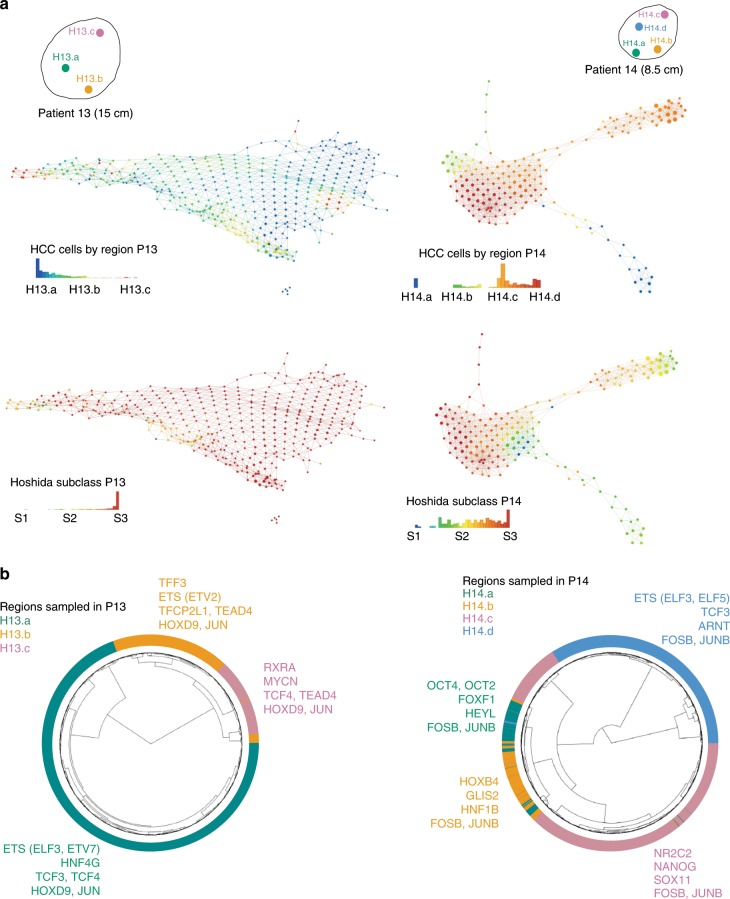
Regulatory network heterogeneity at the single-cell level. **a** Geographic distribution of the multiregional sampling for single-cell RNA-seq. Topology data analysis of HCC-cell population across regions, as visualized with Ayasdi Platform^74^. Cells are labeled based on tumor region and molecular subclass. Each dot represents a node, the size of which corresponds to the number of cells that were clustered to form that node. Lines or edges between nodes indicate they have cells in common. **b** Circular hierarchical clustering of HCC cells based on the activation status of TF derived from regulatory networks using SCENIC^34^ (H13.a: Green; H13.b: Yellow; H13.c: Pink). Source data are provided as a Source Data file.

Our bulk sequencing data suggest a strong impact of cell-type admixture in ITH. Thus, to explicitly examine the HCC ecosystem at the cell-type and gene regulatory network level, we conducted whole lysate (i.e., no previous cell enrichment) single-cell RNA-seq from geographically distant regions in 2 HCC patients. Overall, we profiled 21,143 and 17,410 cells from 3 and 4 tumoral regions in P13 and P14, respectively. Computing the t-SNE plot and labeling cells based on the region they were derived shows that most clusters are contributed by cells obtained from all three regions in P13 (Fig. 6a). Conversely, the t-SNE plot in P14 is more regionally clustered, with cell clusters mainly contributed by distinct single tumor regions. As expected, the majority of cells detected in both patients had hepatocyte lineage. Differentially expressed genes across clusters in P13 (Fig. 6b, Supplementary Data File 4) revealed an ecosystem of cells including hepatocytes (*ALB*, *FGG*), cancer-associated fibroblasts (*ACTA2*, *TAGL*), endothelial (*KDR*, *VWF*), myeloid-derived (*HLA-DQB1*, *CD68*), and sporadic B-cells (*IGJ*, *CD79A*), which is consistent with the lack of immune infiltrate on histological examination in this patient (Supplementary Fig. 1b). Using these same markers, we recapitulated identical cell lineages in P14. However, we did detect another lineage not present in P13, characterized by the overexpression of *GNLY*, *NKG7*, and *CCL5* (Supplementary Fig. 9). *GNLY* is a cytolytic protein produced by activated T and NK cells with lytic activity against tumor cells and microbes^32^. Co-expression of CD3 and GNLY in these cells using immunofluorescence (Fig. 6c), predominantly in region H14.c, confirmed their cytotoxic phenotype. To test the hypothesis that regional variance of HCC-cell expression in P13 is lower than in P14, we first selected HCC cells and labeled them by their enrichment in well-known HCC molecular classes^33^. We found that while most HCC cells in P13 belong to the less aggressive S3 class, in P14 there is a strong representation of all three molecular subclasses, as visually summarized in a topographic data analysis of the expression data (Fig. 7a). We cannot rule out that any of the other cells of the tumor microenvironment detected in these patients (e.g., myeloid-derived, CAFs) could also drive ITH in HCC.

Next, we sought to quantify the regional transcriptional states of HCC cells by inferring regional coexpression networks. The transcriptional state of a cell depends on the underlying gene regulatory network (GRN) resulting from upstream transcription factor (TF) regulation. Technical and biological variance in single-cell expression data (e.g. drop-out noise) typically hampers gene expression network analysis, but recently *cis*-regulatory sequence motif analysis has allowed scoring each cell by GRN activity^34^. We leveraged these techniques to score GRN activity and derive HCC-cell states across the different regions of the same tumor nodule. Focusing only on HCC cells, we quantified the activity of regional HCC GRNs and clustered cells based on similar activation patterns (Fig. 7b). We found that while key regulating TFs were turned on in all regions for each of the patients (FOSB, JUNB in P14; HOXD9, JUNB in P13), there was a remarkably high degree of regionality in TF activation patterns even after regressing out regional biases (e.g., cell yield, number of unique molecular identifiers, Supplementary Data File 5). For example, in the poorly differentiated region H14.a we found a very distinct GRN activation pattern of pluripotency signaling orchestrated by the Yamanaka factor OCT4^35^, as well as an overall enrichment in NOTCH signaling compared to any other region of P14 (Reactome, FDR = 0.05). Similarly, uniquely within H13.a we found consistent activation of cell states enriched in the ETS binding domain, namely in the TEL (e.g., ETV7) and ESE (e.g., ELF3) subfamilies of the general ETS transcription factor family, while in region H13.c cell states were dominated by RXRA and MYCN TF activation. Altogether, these data reveal significant heterogeneity in TF activation status across distant regions within the same tumor nodule.

## Discussion

Using multiregional omics data from 71 samples (*N* = 14 patients) we have unraveled key readouts of ITH in HCC. We detected ITH in 30–40% of treatment-naive HCC encompassing neoepitope burden, TIL burden and clonality, HBV expression and regional gene expression profiles. A deeper characterization of HCC-cell specific ITH using single-cell RNA-sequencing, reveals substantial regulatory heterogeneity. Our finding of significant regional differences in the magnitude of immune infiltrate in HCC confirms previous reports^12^. Moreover, we derived gene expression signatures reflecting intratumoral expression dynamics, which encompass these extremes of clonal evolution and immune infiltration and found that they outperform single-biopsy derived signatures in predicting survival in the TCGA-HCC cohort. Our observations of different regional immune clonal expansions, and bias towards passenger mutation driven neoepitope production, suggest the complexity of the evolving tumor-immune interactions may contribute to the emergence of ITH. Even though we confirm the presence of significant regional chromosomal instability previously reported in other tumor types^3^, this information alone does not completely recapitulate the full scale of molecular ITH in our HCC samples. Simultaneously measuring TIL burden and clonality, regional neoepitope variance, and potential viral cofactor signals using RNA-seq data significantly increases the scope and value of bulk multiregional data in assessing clinically relevant ITH. Our immunogenomic regional data indicate that passenger mutations potentially contribute more to TIL recruitment than driver mutations.

However, our data also imply that the average MHC-I-binding affinity of tumor neoantigens exceeds that of HBV on a per-antigen basis, suggesting that TIL recruitment is primarily tumor induced. Indeed, we also observe a relative lack of correlation between TIL burden and CTAs/HBV expression. Looking at the actual architecture of the TIL response, it might be plausible to define a chronology of key stages of the tumor-immune interaction, namely negative selection, via the formation (early) and dissolution (late) of TLS. We found hot and cold patterns of regional TIL burden^5^. The cold pattern, epitomized by P10, includes virtually zero TIL. This minimal immune selection pressure likely renders tumor progression a composite of clonal evolution and resource/viral-induced constraints. P10 expresses both clonal *TP53* and *CTNNB1* mutations, which have been proposed as mechanisms of immune exclusion in different tumors, including HCC^36^. A similar pattern is also seen in other patients with either *CTNNB1* or *TP53* mutations. The hot pattern is exemplified by P02 and P06. In these tumors, which lack *TP53* or *CTNNB1* mutations, we observe a regional adaptive immune response, suggestively associated with tumor cell de-differentiation and increased chromosomal instability. Along these lines, a recent study of TIL in HCC confirms intratumor T-cell clonal expansion at the single-cell level and reveals a highly complex T-cell ecosystem^37^.

Our survival analysis reveals that regional transcriptional heterogeneity within a single tumor can be high enough to capture survival signals in a large cohort of single-biopsy HCCs. Indeed, our ITH signature, derived from the intratumor differential gene expression of P02, retains independent prognostic value compared to other well-known HCC survival predictors such as the G3 signature^38^. We speculate on the broader implications of a single patient tumor evolution trajectory capturing survival signals in a single-biopsy cohort such as the TCGA. Our ITH signature from P02 includes tumor states spanning from immune cold and well-differentiated tumor regions (regions B, C, and D) to poorly differentiated, weakly immunogenic and immune hot regions (region A). These data suggest that the intratumoral transcriptomic differences in P02 recapitulate the different stages of tumor evolution found in a large cohort of HCC patients across a spectrum of different clinical stages and somatic mutation burden.

At the single-cell level, we examined the substrate of molecular ITH beyond cataloging the regional variance of cell-type admixture, and quantified gene regulatory heterogeneity of HCC cells. Broadly, P13 resembled the cold P10 while P14 had one region with only moderate immune infiltrate (H14.d), preventing a single-cell view of the hot pattern we observed in the bulk data. The single-cell data did nevertheless identify important basic differences between the two patients, with HCC cells in all regions of P13 mostly belonging to one molecular class while belonging to many classes in P14. At the gene regulatory level, we observe more profound differences in TF signaling among the regions of P13 and P14, including pluripotency signaling in poorly differentiated regions. It is remarkable that TF activation state in HCC cells can largely recapitulate what tumoral region they came from, indicating that downstream expression reprogramming of clonal evolution is highly dynamic.

Despite the relatively low number of patients included in our study, this is the largest and most comprehensive analysis of transcriptomic ITH in HCC reported so far. Other limitations of our analysis include the under-sampled TIL representation and the estimates for RNA-seq variant calling, which are contentious in the context of varying tumor purity. Despite our regional somatic mutation overlap, and targeted DNA mutation validation suggest reasonable coverage levels, our sensitivity is probably low. While whole-exome sequencing data in one of our patients confirmed our main conclusions, the difficult question of how to threshold expressed mutations would remain. Another possible limitation relates to the classic question of the accuracy of in silico predictions of neoantigen binding affinity in estimating immune reactivity. For example, a detailed characterization of the tumor-derived ligandome in melanoma using mass spectrometry questioned the ability of these predictions to identify highly immunogenic neoepitopes, particularly for the top 10 predicted binders^16^. Nonetheless, in silico prediction of binding affinity was successfully used in two phase I clinical trials testing personalized vaccination in patients with melanoma^17,39^. Considering that our study is not aiming to identify top binders but rather characterize broad relative shifts in the predicted binding profile within individual tumors, we believe that these potential discrepancies do not have a major impact on our results. Finally, we acknowledge that including MHC class II epitopes into these regional analyses may offer new insights by more fully characterizing regionally varying adaptive immune response (though we note the relative lower predictive accuracy in binding affinity compared to HLA-I^39^). Experimental studies will be required to validate our results of neoantigen and viral immunogenicity and their impact in immune recruitment and cancer clonal composition in HCC.

In conclusion, this study sheds further light on the underlying molecular features of ITH in HCC including the unexpectedly large scale of regulatory heterogeneity of HCC cells. The relevance of quantifying regional differences in cancer-immune interactions is only highlighted by the ongoing pan-cancer revolution in immunotherapy, providing new directions to treatment biomarker discovery.

## Methods

### Human samples and histological evaluation

All patients were enrolled at Icahn School of Medicine at Mount Sinai (ISMMS) and provided informed consent for tissue biobanking. Study was approved by the Mount Sinai IRB (IRB# HS-14-01011) and samples were provided by the ISMMS Tissue Biorepository (IRB# HS-10-00135). All patients had early stage hepatocellular carcinoma (HCC) as per EASL guidelines^40^, and were treatment-naïve prior to resection. Frozen tissue samples were collected allowing for at least 1 cm of distance between each other. Samples were selected from areas without macroscopic evidence of necrosis or hemorrhage. For morphological analysis, sections were cut (5 µm thick), stained with hematoxylin and eosin (H&E), and evaluated by an expert liver pathologist. The histological features evaluated included tumor grade by the WHO (i.e., well, moderately and poorly differentiated), a semi-quantitative evaluation of immune cell infiltrate and steatosis (absent, mild, moderate, and severe), and enumeration of mitotic figures per high-power field (Supplementary Table 1). Degree of fibrosis in the adjacent nontumoral liver was assessed using the METAVIR scoring system^9^.

### Nucleic acid extraction and DNA sequencing

DNA and RNA were extracted using the DNeasy blood and tissue kit and RNeasy mini kit (Qiagen), respectively. RNA quality was assessed with the RNA Integrity Number (RIN) as provided by 2100 Bioanalyzer (median RIN for samples submitted to RNA-seq was 9). The purified DNA was run on a 2100 Bioanalyzer Instrument (Agilent) for size estimation, and its concentration was measured by fluorometric quantitation using Qubit (ThermoFisher). All targeted DNA sequencing identified somatic mutations predicted as damaging by Poylphen or SIFT and were above 5% VAF were subjected to Sanger sequencing for validation. We used the following criteria for validation of RNA-seq mutation calls with Sanger sequencing: (1) Somatic mutations; (2) predicted as damaging by Polyphen or SIFT, (3) Read depth greater than 10; (4) VAF greater than 40%; (5) recurrent among multiple tumor regions. Primers used for Sanger sequencing are listed in Supplementary Table 5. For Sanger sequencing, each PCR product was assessed on a 1.5% agarose gel, sequenced in both directions using BigDye Terminator Cycle-Sequencing Kit (Macrogen) and loaded on an ABI PRISM 3730xl DNA analyzer. Sequences were analyzed using the Applied Biosystems’ sequencing analysis software with the KB base-caller. Targeted next generation DNA sequencing was performed for all exons of a panel of 58 genes frequently mutated HCC genes (Supplementary Table 6). Indexed Illumina NGS libraries were prepared from tumor and nontumor adjacent tissue (P01-5) or peripheral blood mononuclear cells when available (P06-10). Sequence captures were carried out using the Biotinylated custom baits of Agilent SureSelect oligo pool (Agilent Cat #5190-4808). DNA targeted sequencing data from P6-P10 were recently reported, including detailed methodology of library preparation, sequencing, and data analysis^41^. Whole-exome sequencing analysis of patient 10 was performed on 125 bp paired end reads using an Illumina Nextseq 500 platform (Nextseq High output flow cell, 300 cycle baits). Libraries were constructed using the SureSelect XT low input with V7 baits (Protocol Version B1) following the standard protocol. WES data processing was performed using a custom nextflow pipeline that is available on GitHub (https://github.com/losiclab/exoseq). Raw reads were trimmed and aligned to the hg38 reference genome using trim-galore^42,43^, bwa mem^44^, and samtools^45^, respectively. Duplicate reads were then marked using picard MarkDuplicates, and bam quality scores were recalibrated for known technical bias using GATK4 base recalibration^46^. Quality control metrics were compiled using fastqc2 for raw reads and picard CollectMultiMetrics for aligned reads. Somatic variants were called using Mutect2^46–48^ in WES tumor-normal matched data, with the intervals parameter set to all coding regions, and with 1000 genomes as the germline resource. Variants were filtered for quality control using GATK FilterMutectCalls with default parameters. Only variants with a VAF > 5% were retained for further analysis. Variants were annotated with Annovar (version 2019Apr09).

### DNA copy number analysis

Hybridizations were performed at the Genomics Core Facility of the ISMMS using the high-resolution HumanOmni2.5-8 Beadchip genotyping arrays (Illumina). Adjacent nontumoral tissue or peripheral mononuclear blood cells (for P07, since no adjacent nontumoral tissue was available for this patient) were used as controls. Copy number variation was studied at the level of allele-specific variation (ASCAT version 2.1^10^) and at whole copy number variation (Circular Binary Segmentation, CBS, relying exclusively on log R Ratios of cases vs. controls). We used ASCAT to dissect the allele-specific copy number alterations, while simultaneously estimating and adjusting for both tumor ploidy and non-aberrant cell admixture. To analyze focal events of the copy number alteration (CNA) profiles, we used as input the average Log R Ratios per segment obtained from CBS.

### Immunofluorescence staining

Frozen histological sections were immersed in pre-cooled methanol (−20 °C) for 15 min. Blocking was performed for 1 h at room temperature with a solution composed of 1X TBS, 10% BSA and 0.3% Triton-X. Sections were incubated overnight (4 °C) with primary antibodies against CD-3 (dilution 1:50, DAKO A0452), CD-20 (dilution 1:200, DAKO M0755), granulysin (dilution 1:100, Santa Cruz sc-271119), and PNAD (dilution 1:200, BD Biosciences 553863). AlexaFluor® 488 (dilution 1:200, Invitrogen A21121 and A21212), AlexaFluor® 546 (dilution 1:200, Invitrogen A11030), and AlexaFluor® 594 (dilution 1:200, Invitrogen A11037) secondary fluorescent antibodies were applied for 1 h at room temperature. Nuclei were labeled with DAPI (dilution 1:1000, Invitrogen D1306) and slides were mounted with Fluoromount-G® (SouthernBiotech). Stained slides were evaluated using a Nikon Eclipse NI microscope and a Zeiss Axio Observer 7 with appropriate filters.

### RNA sequencing

RNA-seq was conducted on poly-A enriched RNA, 100 bp single reads using an Illumina HiSeq2500 instrument. Among the tumor regions analyzed with RNA-seq, we also included a technical replicate of region A of P02 (i.e., H2.a). Libraries were constructed using the TruSeq RNA Library Prep Kit v2. Raw sequencing reads were mapped to the GRCh38 reference genome (USCS) using STAR (version 2.4.2g1)^49^. Aligned reads were mapped to GRCh38 genetic features using featureCounts from the subRead package^50^ with default settings, with a median coverage of 30 million mapped reads per region.

### HLA typing and expression

Raw sequencing reads were re-mapped to all known HLA-I alleles using a 4-step approach. First, a low-stringency mapping was performed using razers3 to identify HLA-matching reads^51^. If there were more than 10,000 such HLA reads, they were next randomly down sampled to produce a more manageable, smaller output file (with a maximum of 10,000 reads). For step three, Optitype (version 1.0)^52^ was used on this low-stringency, potentially down-sampled HLA-specific, razers3 output to consensus call the HLA alleles. Briefly, this method finds an allele combination that maximizes the number of reads they explain. Finally, the overlap of reads mapping to distinct alleles was quantified by assigning fractions of read support to each allele using a custom script that operates only on the high-stringency mapping output of Optitype.

### RNA variant calling and putative neoantigen calling

Mapped RNA-seq reads were subject to splitting, trimming, local indel realignment, and base-score recalibration pre-processing with the IndelRealigner and TableRecalibration tools from GATK^53^ under the GATK Best Practices for RNA-seq paradigm. Mutect (version 2.0)^48^ was then used to compute the regional somatic mutation burden in the following fashion. For each patient, all reads from adjacent nontumoral regions were combined to form an effective normal against which tumor regions were tested for somatic mutations. Somatic calls from mutect with fewer than 10 supporting variant reads were not considered. The technical replicate for P02, region A, was removed due to a failure in the GATK base quality recalibration model. To predict neoantigen and associated epitope burden, we used Topiary (Rubinsteyn and Nathanson, https://github.com/hammerlab/topiary) to call mutation-derived cancer T-cell epitopes from somatic variants, tumor RNA expression data, and patient class I HLA type. This tool matches mutations with gene annotations, filters out non-protein coding changes, and finally creates a window around amino acid changes, which is then fed into NetMHCCons for each patient HLA allele across tiles of 9-12 amino-acid in length^20^. Given that HLA-I processes neoantigens by degradation to non-conformational 8-11 amino-acid residues, we only included those sizes and excluded neoepitopes with mutations obscured to T-cells within HLA-I binding pockets. In the case of frameshift mutations, in principle this window starts from the mutation minus the length of the peptide up to the first stop codon. To compare pairs of empirical cumulative density (ECDF) of binding affinities between regional tumor neoantigens within a patient, we used a one-sided Kolmogorov-Smirnov test. The alternative hypothesis is that one ECDF is shifted to lower binding affinity compared to the other. Since the high binding affinity modes of the distribution are essentially noise from the netMHCCons predictions, the one-sided test is mainly sensitive to differences in the low binding affinity tail, which contains putative neoantigens. A low p-value indicates that one distribution is shifted towards higher binding affinity (i.e., higher putative immunogenicity) compared to the other.

### T/B cell receptor sequencing and inference

*DNA*: T cell receptor beta chain CDR3 regions were sequenced by ImmunoSeq (Adaptive Biotechnologies), with primers annealing to V and J segments, resulting in amplification of rearranged VDJ segments from each cell. Clonality and richness values were obtained through the ImmunoSeq Analyzer software. Differential abundance analysis was assessed using Fisher’s exact test and a beta-binomial method to increase stringency, as previously described^54,55^, to identify clones that were significantly expanded in different regions of the same tumor nodule.

*RNA*: Mapped RNA-sequencing reads were used to allelotype (MHC class-I loci) each patient, estimate the putative TIL burden per patient by profiling TCR and BCR sequences with MiXCR^56^, and normalizing by patient library size. Generally the strategy for this class of algorithms can be summarized in several key steps, namely stringent pre-processing (including using frequency-based corrections for PCR artifacts and other sequencing errors), basic corrections for allelic differences between patients, and identification of deletions and insertions prior to alignment to receptor sequence without intronic sequence for the case of RNA-seq reads. In detail, after pre-processing, within MiXCR the basic workflow starts with alignment, where sequencing reads are aligned to spliced reference V, D, J, and C genes of T or B cell receptors. After that there is a partial assembly step whereby overlapping sequencing reads (which are expected in nontargeted or RNA-sequencing reads) are joined into sufficiently long CDR3-containing contigs for downstream analysis. We set the minimal overlap to be five base pairs and the length of the kmer taken from the VJ junction for overlap search to be 12 base pairs. We also go through a procedure of extension for imputation of higher quality germline sequences from well-trimmed TCR sequencing reads. To quantify VDJ expression per sample, only reads that supported these CDR3 contigs were counted and then subsequently normalized by the total library size of that RNA-seq sequencing run, as are the relative number of reads supporting each distinct CDR3 contig sequence assembled.

### HBV expression, integration, and antigen binding affinity

Raw RNA-sequencing reads that did not map to the GRCh38 reference genome was assembled into contigs using Trinity (using –no_run_chrysalis –no_run_butterfly flags, which effectively only invokes Inchworm) to perform greedy kmer-21 contig assembly. Contigs with a sufficiently high entropy (to exclude homopolymer sequences), at least 100 bp long and supported by at least 20 reads were retained for further analysis. Contigs were BLASTed (BLAST version 2.2.26 + ^57^) to HBV sequence^58^ and all contigs with bitscore > = 100 were retained. Contig expression was computed using the RPKM summary statistic defined by the number of reads per contig scaled by the product of the total number of unmapped reads for that sample and the contig length. The viral antigen burden and predicted immune binding affinity of HLA/antigen ligand pairs were estimated using the following procedure. First we selected the BLAST contig mappings that maximized the bitscore, a logarithmically rescaled version of the contig raw alignment score that is independent of the size of the search space, for each sample. This defined which reference HBV genome was ‘expressed’ in that sample. We then took that specific HBV genome in its entirety and first computed the longest open reading frames using TransDecoder.LongOrfs and then predicted likely coding regions (CDS) using TransDecoder.Predict^59^ at default settings. Importantly, this means that all ORFs shorter than 300 aa are excised. Aggregating all of the final candidate ORF regions for each sample-specific HBV genome, we fragmented each into overlapping fragments ranging from 9 to 12 aa in length and computed the class I HLA binding affinity using NetMHCCons^20^ for each fragment-patient class I allele combination. Viral integration sites were found by computing putative fusion transcripts between a faux 25th chromosome and the regional bitscore-maximizing HBV genome strain as above. Briefly, we examined the chimeric alignments from the initial regional STAR alignments and post-filtered them with an emphasis on precision using STARChip (version 1.1)^60^.

### Regional expression variance

To account for regional gene expression changes, we carried out statistical tests for differential expression across all combinations of regions within a given patient by testing the null hypothesis that the logarithmic fold change (LFC) between regions for a given gene’s expression is zero. For patients with three or more regional samples, we compared all unique regional combinations building from 2 × 1 comparisons. In order to facilitate gene ranking, stable effect size estimation, and variance sharing across genes among samples we used DESeq2^61^ to model the dependence of the dispersion of the count data on the average expression strength overall of the samples in the comparison. Since all comparisons were between samples on the same genetic background, tissue type, and sequencing run, we simply imposed a more stringent false discovery rate (FDR) of 1% to account for the inherent lack of power of these statistical tests. Gene Set Enrichment Analysis (GSEA)^62^ was used to determine if a gene list composed of expressed CTAs^26^ shows cumulative changes in expression across our ITH dataset. We performed pre-ranked GSEA using the java implementation downloaded from the Broad Institute webpage. Genes were ranked by differential expression between tumor and adjacent samples (determined separately for each patient). Enrichment scores were determined from a running sum statistic, when the statistic is at the maximum deviation from zero.

### Analyses on the TCGA

Mutation Annotation Files (MAF) and RNA-Seq FASTQs for the TCGA dataset (LIHC cohort) were downloaded from the National Cancer Institute’s GDC Data Portal (https://portal.gdc.cancer.gov/) for HCC patients. Matched clinical data were downloaded from the cBioPortal (http://www.cbioportal.org/). RNA-Seq data were aligned to hg38 with STAR (v2.5.1b) in two-pass mode. Gene counts for Gencode v23 (www.gencodegenes.org) gene annotations were generated using featureCounts. Read counts underwent TMM normalization and logCPM transformation using voom^63^.

### Single-cell RNA-seq

Tissue was collected in 5 ml of RPMI media. Further disaggregation of tissue into a single-cell solution for sequencing was completed using the MACs tumor dissociation kit with the standard tough tumor protocol. Briefly, the MACs tumor dissociation kit enzyme mix (300 μl) was added to each sample. Next, samples were put into the gentleMACs Dissociator and ran through the tough tumor program. The cell suspension was then applied to a 70 um cell strainer. Cells were pelleted and resuspended in PBS. Next, the suspension was treated with red blood cell lysis solution for 10 min, diluted in PBS, pelleted and resuspended in 3 ml PBS. Cells were diluted 1:2 in trypan blue prior to counting. The resulting single-cell suspension was diluted to a concentration of 1000 live cells/μl from which 10 μl was used as input for the Chromium^TM^ Single Cell 3’ Protocol as the following describes.

The single-cell chip loading, GEM generation & barcoding, post GEM-RT & cDNA amplification, and library construction were performed according to the Chromium^TM^ Single Cell 3’ Protocol - Chemistry v2. For GEM generation an input of 10,000 cells total, at 1000 cells/μl density, was targeted for each sample, with a target cell recovery of 6000 cells. Library construction, enzymatic fragmentation, End-repair and A-tailing were performed as follows: pre-cool block at 4 °C hold, fragmentation at 32 °C for 5 min; End repair and A-tailing 65 °C for 30 min and held at 4 °C. Post reaction cleanup was performed, followed by adaptor ligation. Adaptor ligation incubation was done at 20 °C for 15 min. Post adaptor ligation cleanup was then performed, followed by sample index PCR with the following parameters: 98 °C for 45 sec; followed by 14 cycles: 98 °C for 20 sec; 54 °C for 30 sec; and 72 °C for 20 sec; followed by 72 °C for 1 min and held at 4 °C. Quantification of the constructed libraries was evaluated using Qubit dsDNA HS Assay Kit (Thermo Fisher), Agilent cDNA High Sensitivity Kit, and Kapa DNA Quantification Kit for Illumina platforms, following the manufacturer’s instructions. Generated libraries were sequenced on the Illumina HiSeq2500, using the paired-end 2 × 125 bp sequencing protocol. Sequencing run parameters were setup according to version 2 chemistry, the number of cycles for each read as follows: Read 1: 26 cycles, i7 index: 8 cycles, i5 index: 0 cycles and Read 2: 98 cycles.

An analysis of the single-cell RNA-seq data was done with the package Seurat (version 2.1)^64^. Initial filtering steps removed all the cells with fewer than 200 genes or a percentage of mitochondrial reads higher than the third quartile in our samples. Reads were normalized, scaled and adjusted for total amount of expression (nUMI) and the percentage of mitochondrial reads using linear regression. Next we computed and clustered the cells with a graph-based algorithm for modularity optimization using the 10 principal components of the normalized expression matrix, computing marker genes for each cluster^65^. Malignant hepatocytes were defined using a reported gene signature derived from human HCC single cells^66^. We also applied nonlinear dimensionality reduction techniques like t-SNE^67^ and topological data analysis (TDA, Ayasdi Platform). Prediction for the molecular classes S1-S3^33^ at the single-cell level was done using permutation tests^68^.

We used the SCENIC workflow (version 1.0)^34^, which consists of three steps. First, TF-directed coexpression networks are learned from the batch corrected, variance stabilized, single-cell RNA expression data using the random forest based approach GENIE3, which allows for nonlinear gene-gene contributions to a particular TF association. To filter these TF- coexpression modules, each was subjected to a cis-regulatory motif analysis using RcisTarget (SCENIC) and only modules with a highly significant motif enrichment (*P* < 0.01) were retained for further analysis and pruned of indirect targets lacking motif enrichment. These filtered TF-coexpression module pairs, called regulons, were then projected onto the ranks of expressed genes for each cell and compared using the AUCell routine (SCENIC). AUCell uses a cumulative criterion to determine if a critical subset of the regulon gene set is enriched at the top percentile of expression in each cell. Our results do not depend greatly on any reasonable choice of this threshold.

### Survival analysis

We used Kaplan-Meier curves and log-rank test to evaluate the impact of mutation load, number of tumor clones and the ITH signature on patient’s outcome in the TCGA dataset. We first conducted differential gene expression between all tumor regions in those patients with at least three regions sampled. The *p*-values for this analysis were computed from the standard parametric differential expression test assuming a negative binomial count distribution (DESeq2^61^), adjusted by the Benjamini-Hochberg procedure. Using these genes, we created a PCA plot and used the first five principal components (i.e., eigenvectors of covariance matrix) to evaluate their prognostic impact in the TCGA-HCC dataset. We computed the integrated Brier Score to evaluate the prognostic impact of each of these gene sets^69^, employing nested-cross validation to simultaneously learn ITH signatures and estimate extra-sample (generalization) error (as shown in Supplementary Fig. 8). Explicitly, we carried out the following procedure:

1. Randomly divide the LIHC HCC subset of TCGA data into *K* = 3 folds with approximately equal numbers of survival events.
2. Outer loop: For each ki within the λ*K* folds we performed:
  - Set *K*i-fold as the test set.
  - Perform an elastic-net penalized regression with the set of all intratumoral gene expression differences (called gene expression gradients) in tumors with at least three regions sampled tested as ITH hyperparameters (**I**), which acts as an automated hyperparameter learning, on the remaining *K* − 1 folds.
  - For a given specific learned gene expression gradient in I
    i. Inner loop: For each *K*_j in remaining *K* − 1 folds:
      1. Set fold *K*j as validation set
      2. Train new elastic-net on *remaining K* -2 folds using leave-one-out cross-validation to obtain overall penalization factor λ* (λ*(1 − α)*L2 penalty + α*L1 penalty)), 0 < = α < = 1, λ > = 0
      3. Evaluate model performance on fold *K*j, extracting putatively optimal (minimal) and parsimonious (1se)  λ
    ii. Calculate average performance of ITH parameter *setting I over K* - 2 folds
  - Train model that had optimal-performing gene expression gradient I from inner loop *over K* − 1 folds
  - Evaluate performance via *K* = 10 cross-validation on fold *K*i by computing Lebesgue integrals over discontinuous Brier scores to find Integrated Brier Score (IBS)
3. Average IBS-scores (performance) of test models over *all K* folds.
4. Report averaged IBS score and compare to .632 + bootstrap-resampling estimate previously obtained.

We then repeated the whole nested-cross-validation (nested-cv) procedure, steps 1–4, over a range of penalty-mixing α-choices ((ridge-like) 0.1 < α < 1 (lasso)) in elastic-net in order to hedge against an arbitrary or self-serving choice of α. In other words, we also averaged the entire nested-cv procedure over α. We used a procedure of variance ranking^30^ to select the minimum number of genes required to retain the predictive power of the ITH signature. We selected the genes identified in the top 5% by this procedure, which resulted in 363 genes (i.e., 140 upregulated in H2.a vs the other regions of patients 2 and 223 downregulated in H2.a vs the other regions). We used the Nearest Template Prediction method^70^ to determine which patients in the TCGA-HCC dataset had a significant enrichment of the ITH signatures. To control for optimism^71^, we computed the model discrimination indices for the ITH signature as well as other known prognostic signatures in HCC^31^. Using the learned ITH signatures we controlled for multiple testing and random patient effects by performing repeated cross-validation to explicitly compute the discrimination indices for ITH and other models (see Supplementary Fig. 8). These analyses, principally nested-cross validation, ensured that neither a random patient nor gene selection effect account for the prediction accuracy of our ITH signature. We conducted multivariate analyses using Cox regression modeling including the ITH signature and other potential correlates of cancer evolution such as DNA-based tumor clonality or mutational burden. We also used our TCGA derived Bayesian gene regulatory network to score the potential deleterious downstream impact of mutations. We intersected each patient’s mutational signature on the network and computed the statistics of nodal and global, averaged topological quantities such as out degree, neighborhood connectivity, and clustering (GSE63898) coefficient^72^ (Supplementary Table 7). The prognostic performance of the ITH signature was also tested in the Heptromic dataset, consisting of 228 HCC patients treated with resection for which whole-transcriptome data are already available^73^.

### Reporting summary

Further information on research design is available in the Nature Research Reporting Summary linked to this article.

## Supplementary information

Supplementary Information

Description of Additional Supplementary Files

Supplementary Data 1

Supplementary Data 2

Supplementary Data 3

Supplementary Data 4

Supplementary Data 5

Reporting Summary

## Data Availability

Sequence data (i.e., RNA-seq, scRNAseq, DNA targeted, exome sequencing) and genotyping arrays are publicly available through accession numbers: E-MTAB-5905 (source data underlying Figs. 1c, 2a, b, 3a, b, 3d, 4a–c and Supplementary Figs. 2b–c, 3a, b, 4, 5, 7, 8a, b) GSE112271 (source data underlying Figs. 6a, b, 7a, b and Supplementary Fig. 9), E-MTAB-5899 (source data underlying Supplementary Fig. 3c), E-MTAB-8127 (source data underlying Supplementary Fig. 6), E-MTAB-5878 (source data underlying Figs. 1b, 3c), https://clients.adaptivebiotech.com (source data underlying Fig. 2c). RNA-seq and SNP array from patient 5 are not deposited due to lack of patient-specific deposition consent. Mutation Annotation Files (MAF) and RNA-Seq FASTQs for the TCGA dataset (LIHC cohort) were downloaded from the National Cancer Institute’s GDC Data Portal (https://portal.gdc.cancer.gov/) for HCC patients (source data underlying Fig. 5a–d and Supplementary Fig. 8a–c). The Heptromic Cohort expression array data has previously been deposited at gene expression omnibus (GSE63898, source data underlying Supplementary Fig. 8d, e).

## Source Data

Source Data

## Code availability

Custom R code for the analyses included in the study can be obtained by contacting directly Dr. Losic (bojan.losic@mssm.edu).
