# Supplementary material for: Intratumoral heterogeneity and clonal evolution in liver cancer
Source: Nat Commun. 2020 Jan 15;11:291. doi: 10.1038/s41467-019-14050-z (PMC6962317; doi:10.1038/s41467-019-14050-z)
Supplement: Supplementary file 1 — Supplementary Information [file 41467_2019_14050_MOESM1_ESM.pdf]

Supplementary Information for:

Intra-tumoral heterogeneity and clonal evolution in liver cancer

Losic & Craig et al.

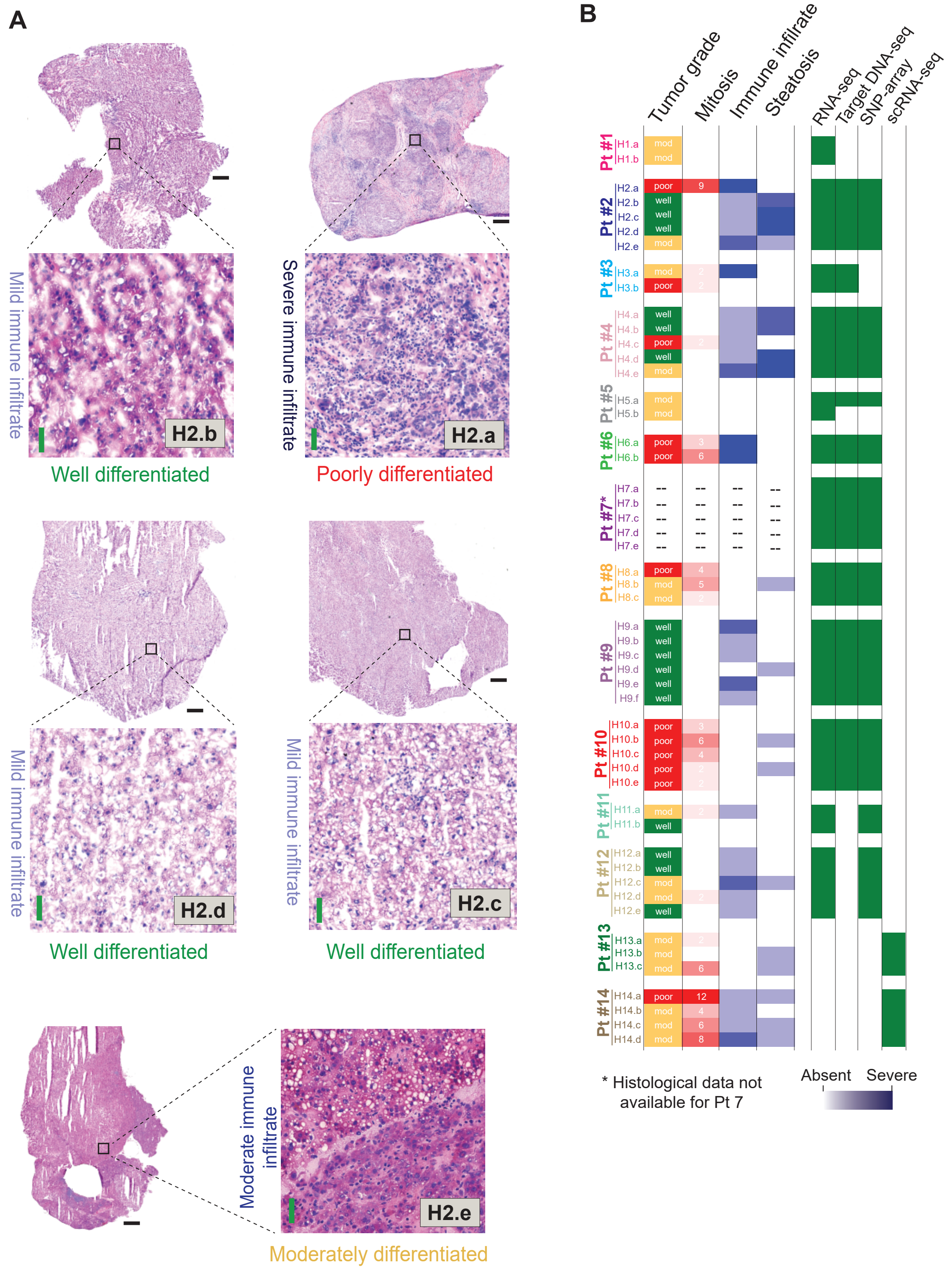

Supplementary Figure 1. **(A)** Representative H&E of the tumor regions in P02 showing phenotypic heterogeneity, both in tumor grade and immune infiltrate (black bars represent 1 mm; green bars represent 50 µm, N=1 experiment). (**B**) Summary of the histological evaluation and genomic profiling of the regions analyzed.

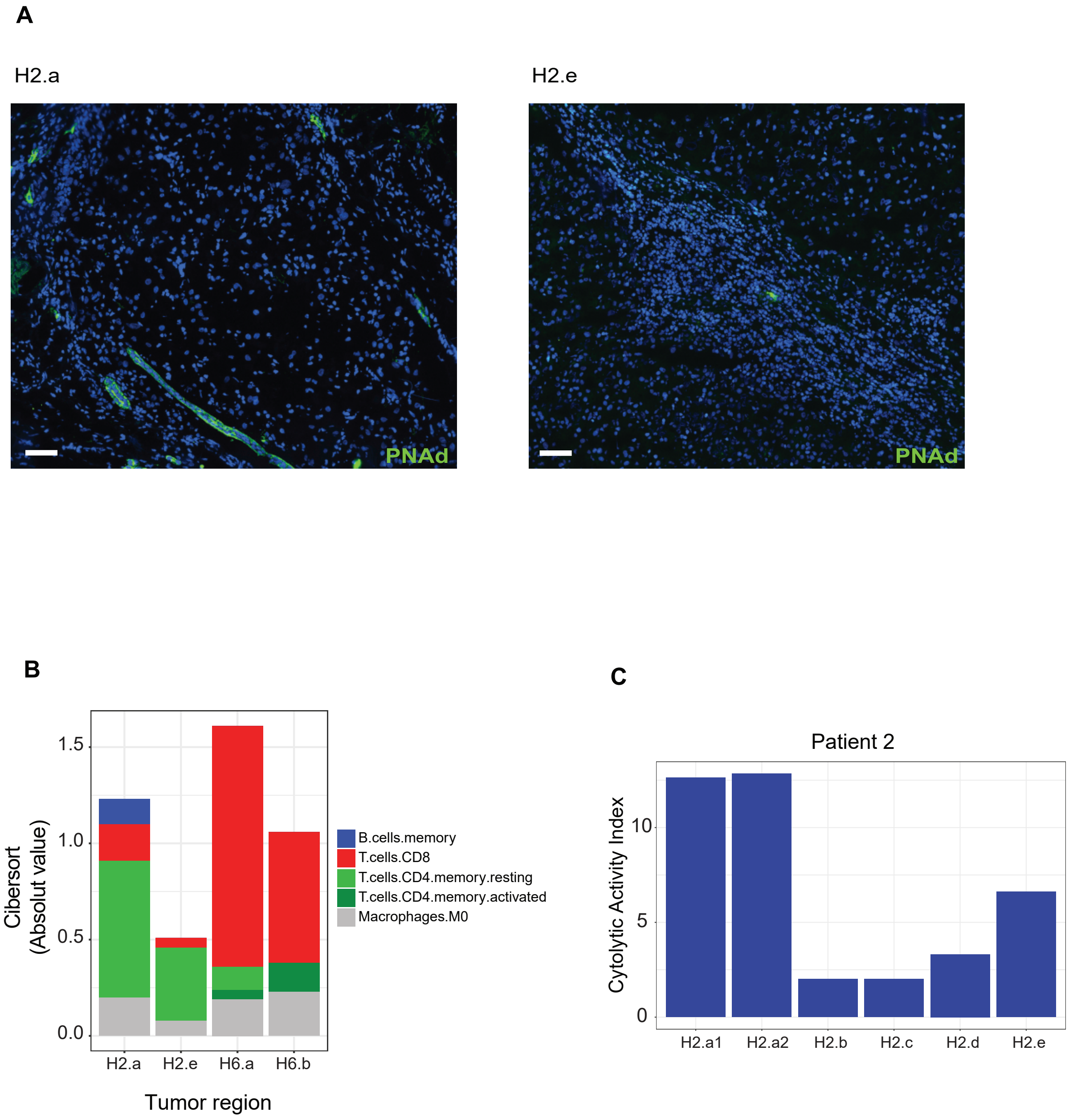

Supplementary Figure 2. (**A**) Immunofluorescence staining of PNAd in regions H2.a and H2.e of patient 2 to detect High Endothelial Venules as part of TLS (Blue: DAPI; Green: PNAd; white bars represent 50 µm, N=3 independent experiments). (**B**) Absolute values for memory B cells, CD8 T cells, CD4 memory resting T cells, CD4 memory activated T cells and M0 macrophages for regions H2.a, H2.e, H6.a and H6.b as determined by Cibersort. (**C**) Cytolytic Activity Index across regions of patient 2. Each bar represents 1 sample.

A

B

C

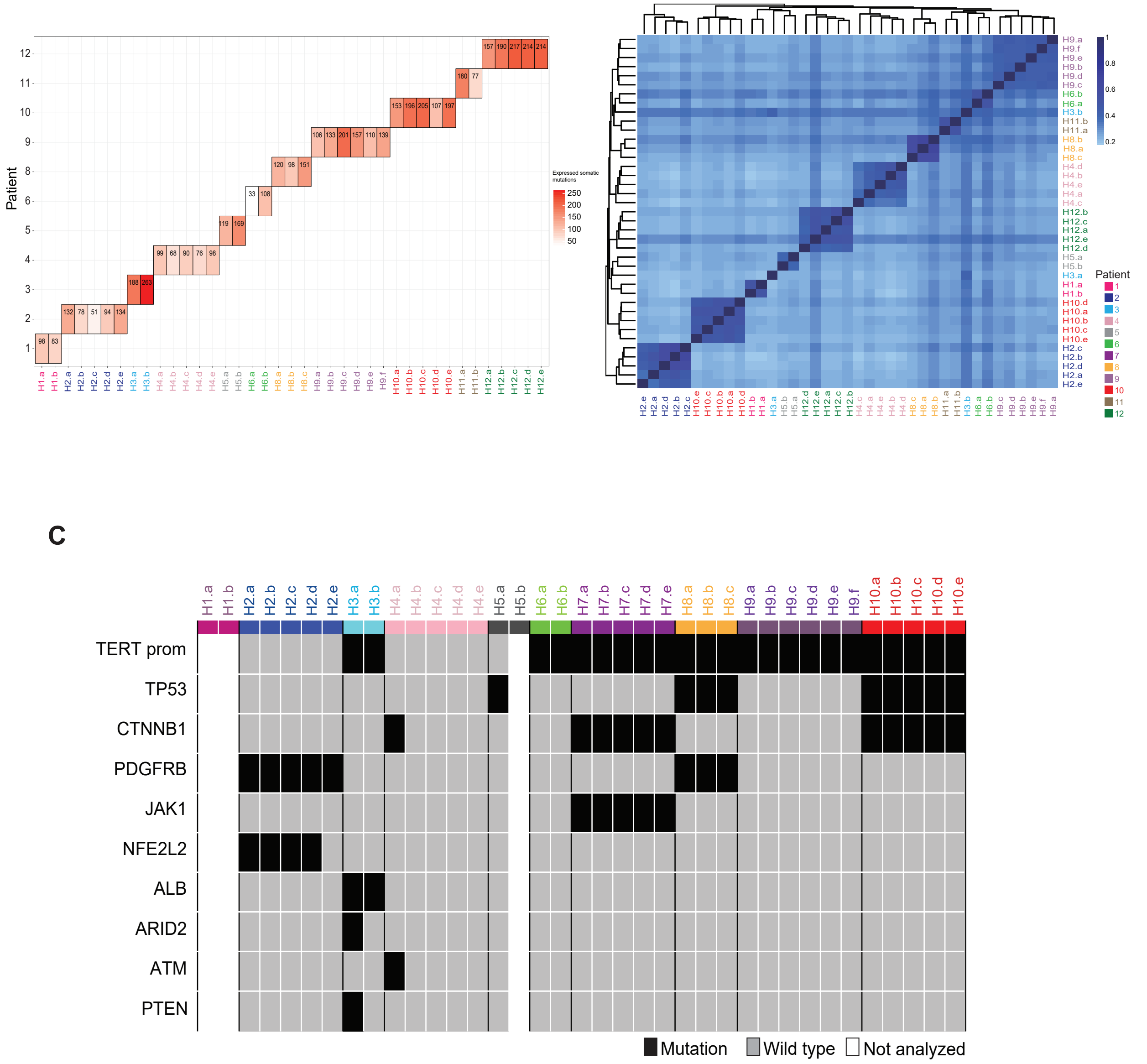

Supplementary Figure 3. (**A**) Number of somatic expressed mutations identified from RNA-sequencing data for each tumoral region. (**B**) Clustering of the overlap of RNA-seq identified somatic mutations for all tumoral regions (blue scale represents Pearson's coefficient values). (**C**) Somatic mutations identified by DNA targeted sequencing. All mutations were called based on a VAF of at least 5% and predicted as damaging by polyphen or SIFT. Source data are provided as a Source Data file.

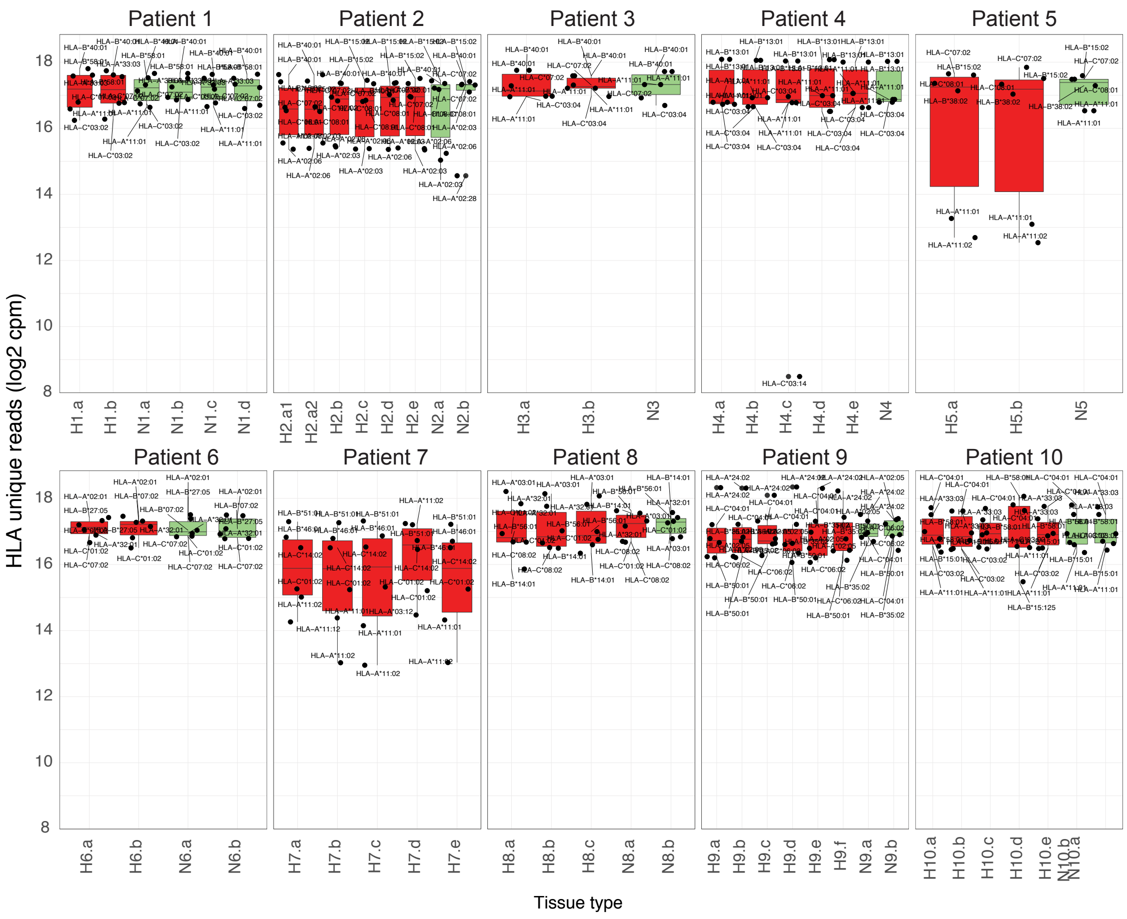

Supplementary Figure 4. Expression of HLA-I alleles (unique reads) across all regions of distinct patients, including non-tumoral adjacent tissue (HCC: Red; Adjacent non-tumoral: Green, allele-specific expression in units of log2 counts per million across N> 100 alleles. For each boxplot, the centre line represents the median. Upper and lower limits of each box represent the 75th and 25th percentiles, respectively. The whiskers represent the lowest data point still within 1.5 × box size of the lower quartile and the highest data point still within 1.5 × box size of the upper quartile. Source data are provided as a Source Data file.

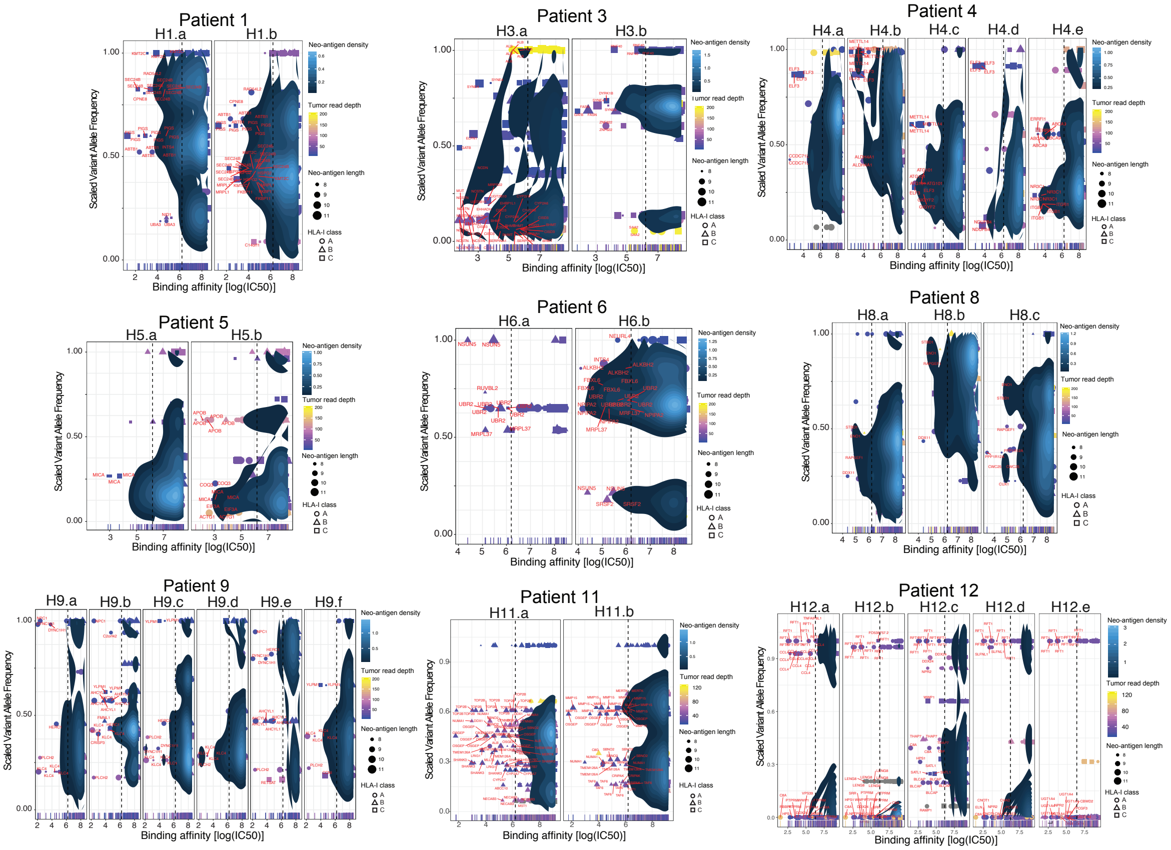

Supplemental Figure 5. 2D density of log-scaled peptide binding affinity as a function of the VAF of somatic mutations across regions of the remaining patients not included in Fig 2. Dotted line depicts 50% inhibitory concentration (ic50)=500 nM (lower ic50 means stronger binding and higher immunogenicity). Source data are provided as a Source Data file.

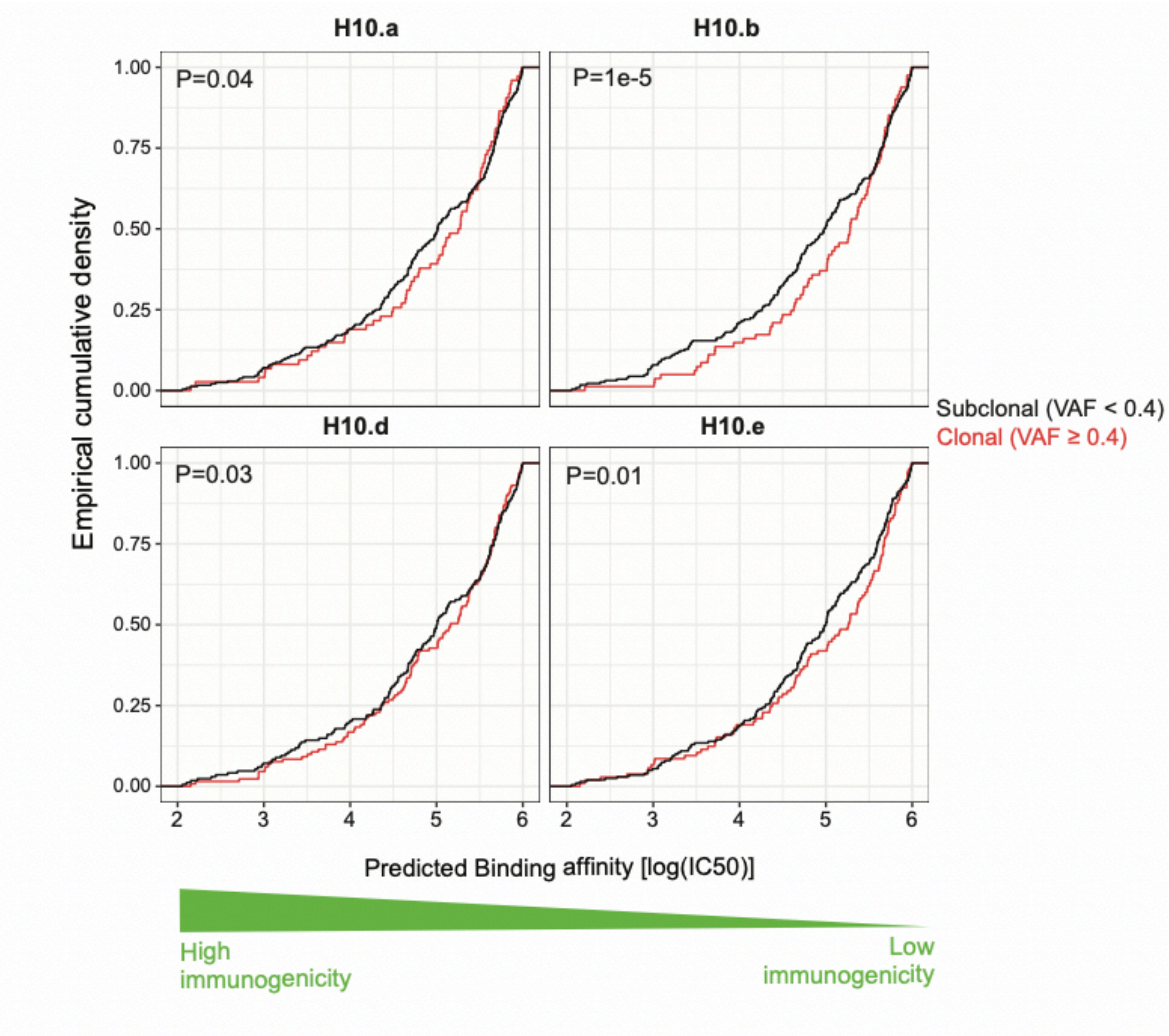

Supplemental Figure 6. Empirical cumulative density plot of log-scaled binding affinity distribution for neo-antigens according to VAF of expressed mutations in each tumoral region of patient 10 determined from orthogonal whole exome sequencing. Kolmogorov-Smirnov test with one-sided alternative hypothesis. P-value is for rejecting the null in favor of the alternative. Source data are provided as a Source Data file.

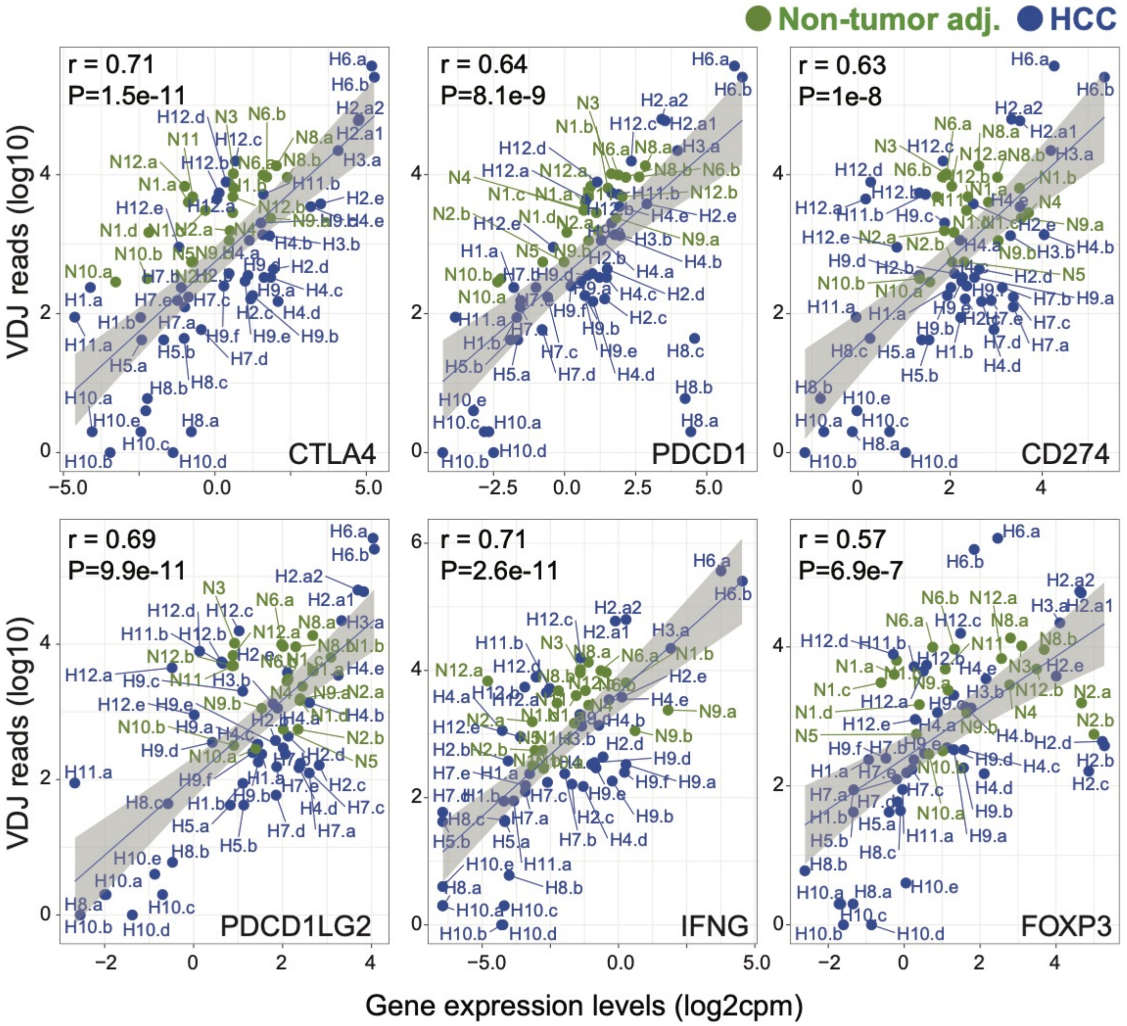

Supplemental Figure 7. Correlation of gene expression of key immune (checkpoint) genes *CD274, CTLA4, IFNG, PDCD1, PDCD1LG2, FOXP3* and VDJ reads, as a proxy for TIL burden (HCC: Blue; Adjacent non-tumoral: Green, N=63 samples). Source data are provided as a Source Data file.

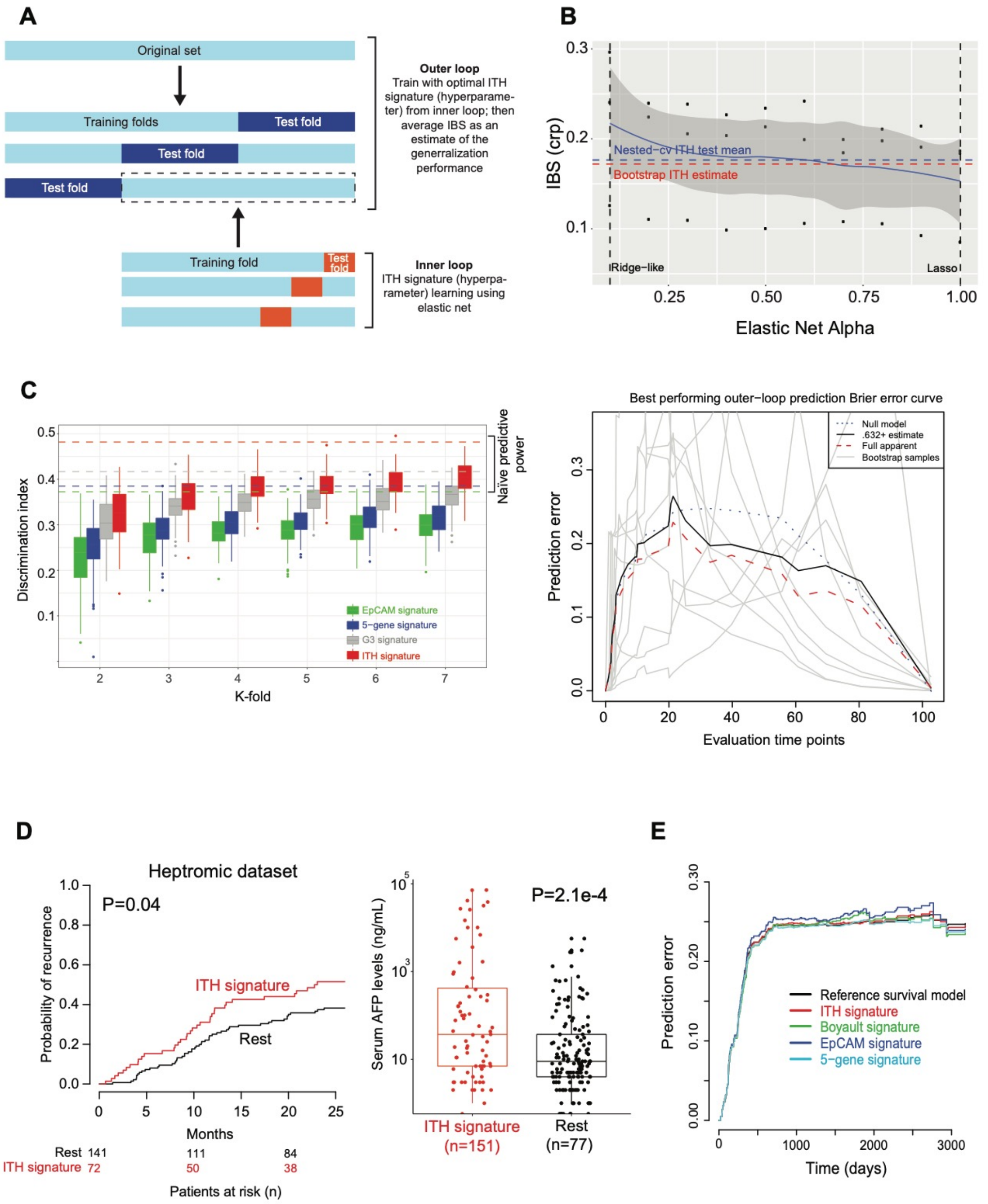

Supplemental Figure 8. (A) Repeated cross validation of fitted survival models. **(B)** Corrected discrimination index as a function of the number of groups of patients that are omitted in the cross-validation simulation (N=500 simulations). The nominal (optimistic) discrimination index is also shown. **(C)** Probability of early tumor recurrence over time for patients in the Heptromic Cohort with or without the ITH signature (Kaplan-Meier (log-rank) test). **(D)** Box plot of Serum AFP levels for patients in the Heptromic Cohort with or without the ITH signature (T-test, two-sided). For each boxplot, the centre line represents the median. Upper and lower limits of each box represent the 75th and 25th percentiles, respectively. The whiskers represent the lowest data point still within 1.5 × box size of the lower quartile and the highest data point still within 1.5 × box size of the upper quartile. **(E)** Prediction error curves of competing parametric Cox proportional hazard models depicting time dependent Brier score for models built from principal components of the G3, EpCAM, 5-gene and ITH signatures. Source data are provided as a Source Data file.

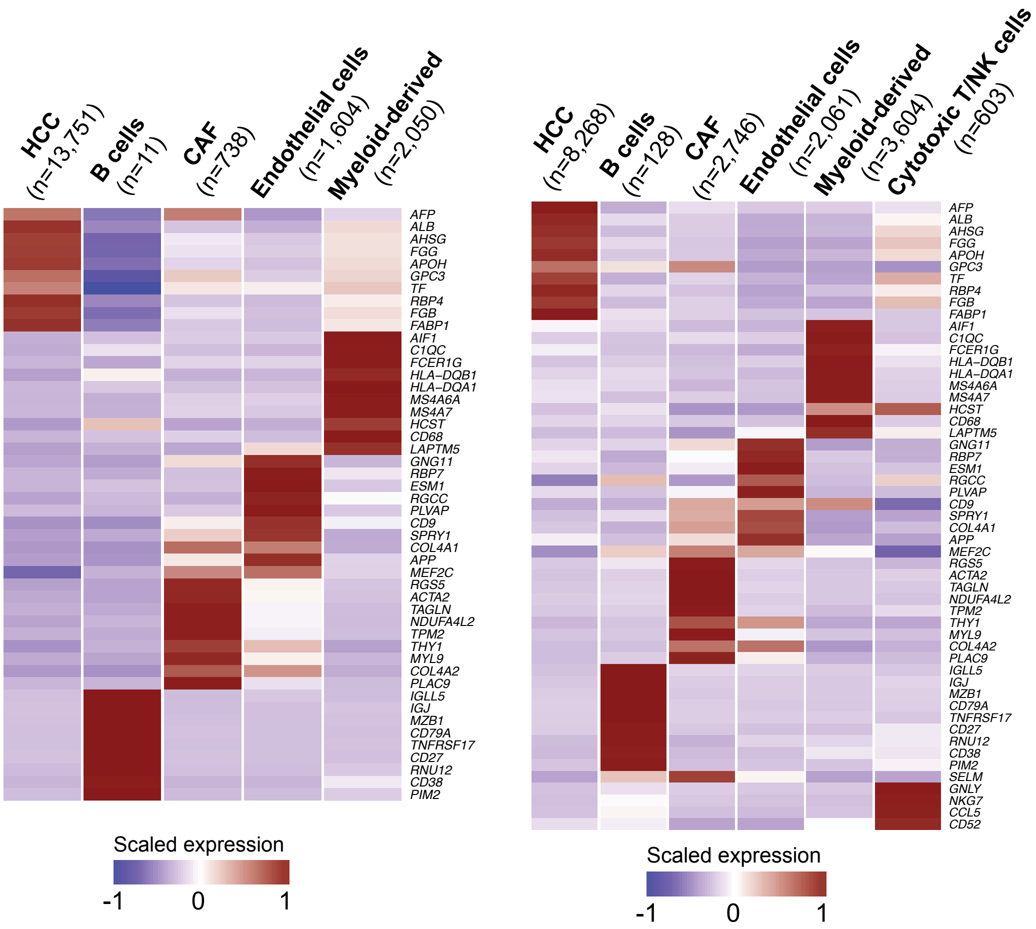

Supplemental Figure 9. Heatmap of differential gene expression in single-cell data across the different cell clusters as per t-SNE plot for patient 13 (left) and patient 14 (right). Source data are provided as a Source Data file.

Supplemental Table 1: Clinical and pathological characteristics of the samples included.

Patient	Gender	Age	Etiology	Size (cm)	Sample	Type	RNA-seq	Tumor grade (WHO)	Immune infiltrate	Tumor cell steatosis	Mitosis (n/10 HPF)	Fibrosis adjacent tissue†
1	Male	59	HBV	4	H1.a	HCC	Yes	Moderately	Absent	Absent	1	NA
					H1.b	HCC	Yes	Moderately	Absent	Absent	1	NA
					N1.a	Adjacent	Yes	NA	NA	NA	NA	Mild (F1-F2)
					N1.b	Adjacent	Yes	NA	NA	NA	NA	Mild (F1-F2)
					N1.c	Adjacent	Yes	NA	NA	NA	NA	Mild (F1-F2)
					N1.d	Adjacent	Yes	NA	NA	NA	NA	Mild (F1-F2)
2	Female	52	HBV	6.5	H2.a	HCC	Yes	Poor	Severe	Absent	9	NA
					H2.b	HCC	Yes	Well	Mild	Moderate	1	NA
					H2.c	HCC	Yes	Well	Mild	Severe (microvesicular)	1	NA
					H2.d	HCC	Yes	Well	Mild	Severe (microvesicular)	1	NA
					H2.e	HCC	Yes	Moderately	Moderate	Mild	1	NA
					N2.a	Adjacent	Yes	NA	NA	NA	NA	Mild (F1-F2)
					N2.b	Adjacent	Yes	NA	NA	NA	NA	Mild (F1-F2)
3	Female	70	HBV	3	H3.a	HCC	Yes	Moderately	Severe	Absent	2	NA
					H3.b	HCC	Yes	Poor	Absent	Absent	2	NA
					N3	Adjacent	Yes	NA	NA	NA	NA	Severe (F3-F4)
4	Male	54	HBV	15.5	H4.a	HCC	Yes	Well	Mild	Moderate	1	NA
					H4.b	HCC	Yes	Well	Mild	Moderate (microvesicular)	1	NA
					H4.c	HCC	Yes	Poor	Mild	Absent	2	NA
					H4.d	HCC	Yes	Well	Mild	Severe	1	NA
					H4.e	HCC	Yes	Moderately	Moderate	Severe	1	NA
					N4	Adjacent	Yes	NA	NA	NA	NA	Mild (F1-F2)
5	Female	61	HBV	6	H5.a	HCC	Yes	Moderately	Absent	Absent	1	NA
					H5.b	HCC	Yes	Moderately	Absent	Absent	1	NA
					N5	Adjacent	Yes	NA	NA	NA	NA	Mild (F1-F2)
6	Male	74	Cryptogenic	10	H6.a	HCC	Yes	Poor	Severe	Absent	3	NA
					H6.b	HCC	Yes	Poor	Severe	Absent	6	NA
					N6.a	Adjacent	Yes	NA	NA	NA	NA	No fibrosis
					N6.b	Adjacent	Yes	NA	NA	NA	NA	No fibrosis
7	Male	58	Cryptogenic	3.5	H7.a	HCC	Yes	NE	NE	NE	NE	NE
					H7.b	HCC	Yes	NE	NE	NE	NE	NE
					H7.c	HCC	Yes	NE	NE	NE	NE	NE
					H7.d	HCC	Yes	NE	NE	NE	NE	NE
					H7.e	HCC	Yes	NE	NE	NE	NE	NE
8	Male	51	HCV	6.5	H8.a	HCC	Yes	Poor	Absent	Absent	4	NA
					H8.b	HCC	Yes	Moderately	Absent	Mild (microvesicular)	5	NA
					H8.c	HCC	Yes	Moderately	Absent	Absent	2	NA
					N8.a*	Adjacent	Yes	NA	Mild	Absent	1	Severe (F3-F4)
					N8.b	Adjacent	Yes	NA	NA	NA	NA	Severe (F3-F4)

9	Male	79	Cryptogenic	13.5	H9.a	HCC	Yes	Well	Moderate	Absent	1	NA
					H9.b	HCC	Yes	Well	Mild	Absent	1	NA
					H9.c	HCC	Yes	Well	Mild	Absent	1	NA
					H9.d	HCC	Yes	Well	absent	Mild	1	NA
					H9.e	HCC	Yes	Well	Moderate	Absent	1	NA
					H9.f	HCC	Yes	Well	Mild	Absent	1	NA
					N9.a	Adjacent	Yes	NA	NA	NA	NA	No fibrosis
					N9.b	Adjacent	Yes	NA	NA	NA	NA	No fibrosis
10	Male	75	HBV	5	H10.a	HCC	Yes	Poor	absent	Absent	3	NA
					H10.b	HCC	Yes	Poor	absent	Mild (microvesicular)	6	NA
					H10.c	HCC	Yes	Poor	absent	Absent	4	NA
					N10.a	Adjacent	Yes	NA	NA	NA	NA	Severe (F3-F4)
					H10.d	HCC	Yes	Poor	Absent	Mild (microvesicular)	2	NA
					H10.e	HCC	Yes	Poor	Absent	Absent	2	NA
					N10.b	Adjacent	Yes	NA	NA	NA	NA	Severe (F3-F4)
11	Male	61	HCV	3.5	H11.a	HCC	Yes	Moderately	Absent	Absent	2	NA
					H11.b	HCC	Yes	Well	Mild	Absent	1	NA
					N11	Adjacent	Yes	NA	NA	NA	NA	Severe (F3-F4)
12	Female	68	HCV	13.5	H12.a	HCC	Yes	Well	Mild	Absent	1	NA
					H12.b	HCC	Yes	Well	Mild	Absent	1	NA
					H12.c	HCC	Yes	Moderately	Moderate	Mild (microvesicular)	1	NA
					H12.d	HCC	Yes	Moderately	Mild	Absent	2	NA
					H12.e	HCC	Yes	Well	Mild	Absent	1	NA
					N12.a	Adjacent	Yes	NA	NA	NA	NA	Mild (F1-F2)
					N12.b	Adjacent	Yes	NA	NA	NA	NA	Mild (F1-F2)
13	Male	67	Cryptogenic	15	H13.a	HCC	scRNA-seq	Moderately	Absent	Absent	2	NA
					H13.b	HCC	scRNA-seq	Moderately	Absent	Mild	1	NA
					H13.c	HCC	scRNA-seq	Moderately	Absent	Mild	6	NA
14	Female	66	HBV	8.5	H14.a	HCC	scRNA-seq	Poor	Mild	Mild	12	NA
					H14.b	HCC	scRNA-seq	Moderately	Mild	Absent	4	NA
					H14.c	HCC	scRNA-seq	Moderately	Mild	Mild	6	NA
					H14.d	HCC	scRNA-seq	Moderately	Moderate	Mild	8	NA

* N8.a had contamination with tumor cells accounting for less than 10% of total cells in this sample (confirmed on H&E histological examination)

† Fibrosis stage as per METAVIR scoring system

NA: Not applicable

NE: Not evaluated

Supplemental Table 2: DNA targeted deep sequencing and Sanger validation

Sample code	Patient	Location and variant	Gene code	Consequence	Gene	SIFT prediction	Polyphen prediction	Validated by sanger
H2.a	2	2:177234076 C/A	ENSG00000116044	missense variant	*NFE2L2*	deleterious (0)	probably damaging (1)	Not tested
H2.b	2	2:177234076 C/A	ENSG00000116044	missense variant	*NFE2L2*	deleterious (0)	probably damaging (1)	Not tested
H2.c	2	2:177234076 C/A	ENSG00000116044	missense variant	*NFE2L2*	deleterious (0)	probably damaging (1)	Not tested
H2.d	2	2:177234076 C/A	ENSG00000116044	missense variant	*NFE2L2*	deleterious (0)	probably damaging (1)	Not tested
H2.a	2	5:150130602 C/T	ENSG00000113721	missense variant	*PDGFRB*	tolerated (0.25)	possibly damaging (0.513)	Yes
H2.e	2	5:150130602 C/T	ENSG00000113721	missense variant	*PDGFRB*	tolerated (0.25)	possibly damaging (0.513)	Yes
H2.b	2	5:150130602 C/T	ENSG00000113721	missense variant	*PDGFRB*	tolerated (0.25)	possibly damaging (0.513)	Yes
H2.c	2	5:150130602 C/T	ENSG00000113721	missense variant	*PDGFRB*	tolerated (0.25)	possibly damaging (0.513)	Yes
H2.d	2	5:150130602 C/T	ENSG00000113721	missense variant	*PDGFRB*	tolerated (0.25)	possibly damaging (0.513)	Yes
H3.a	3	4:73418113 G/A	ENSG00000163631	missense variant	*ALB*	deleterious (0)	probably damaging (1)	No tissue available
H3.b	3	4:73418113 G/A	ENSG00000163631	missense variant	*ALB*	deleterious (0)	probably damaging (1)	Not tested
H3.a	3	10:87933126 C/T	ENSG00000171862	missense variant	*PTEN*	deleterious (0)	probably damaging (1)	No tissue available
H3.a	3	5:1295113 G/A	ENSG00000164362	upstream gene variant	*TERT**			No tissue available
H3.a	3	5:1295113 G/A	ENSG00000164362	upstream gene variant	*TERT**			No tissue available
H4.a	4	11:108244960 A/T	ENSG00000149311	missense variant	*ATM*	deleterious (0)	possibly damaging (0.771)	Yes
H4.a	4	3:41224612 G/A	ENSG00000168036	missense variant	*CTNNB1*	deleterious (0)	possibly damaging (0.997)	Not tested
H5.a	5	17:7674216 C/A	ENSG00000141510	missense variant	*TP53*	deleterious (0)	probably damaging (0.994)	Not tested
H6.b	6	1:26697482 C/A	ENSG00000117713	missense variant	*ARID1A*	deleterious low confidence (0.03)	possibly damaging (0.699)	Yes
H6.a	6	5:1295113 G/A	ENSG00000164362	upstream gene variant	*TERT**			Not tested
H6.b	6	5:1295113 G/A	ENSG00000164362	upstream gene variant	*TERT**			Not tested
H7.d	7	3:41224610 C/A	ENSG00000168036	missense variant	*CTNNB1*	deleterious (0)	possibly damaging (0.64)	No tissue available
H7.a	7	3:41224610 C/A	ENSG00000168036	missense variant	*CTNNB1*	deleterious (0)	possibly damaging (0.64)	Yes
H7.b	7	3:41224610 C/A	ENSG00000168036	missense variant	*CTNNB1*	deleterious (0)	possibly damaging (0.64)	Yes
H7.c	7	3:41224610 C/A	ENSG00000168036	missense variant	*CTNNB1*	deleterious (0)	possibly damaging (0.64)	Yes
H7.e	7	3:41224610 C/A	ENSG00000168036	missense variant	*CTNNB1*	deleterious (0)	possibly damaging (0.64)	Yes
H7.d	7	1:64839716 A/G	ENSG00000162434	missense variant	*JAK1*	deleterious (0)	probably damaging (0.996)	No tissue available
H7.a	7	1:64839716 A/G	ENSG00000162434	missense variant	*JAK1*	deleterious (0)	probably damaging (0.996)	Yes
H7.b	7	1:64839716 A/G	ENSG00000162434	missense variant	*JAK1*	deleterious (0)	probably damaging (0.996)	Yes
H7.c	7	1:64839716 A/G	ENSG00000162434	missense variant	*JAK1*	deleterious (0)	probably damaging (0.996)	Yes
H7.e	7	1:64839716 A/G	ENSG00000162434	missense variant	*JAK1*	deleterious (0)	probably damaging (0.996)	Yes
H7.a	7	5:1295135 G/A	ENSG00000164362	upstream gene variant	*TERT**			Not tested
H7.b	7	5:1295135 G/A	ENSG00000164362	upstream gene variant	*TERT**			Not tested
H7.c	7	5:1295135 G/A	ENSG00000164362	upstream gene variant	*TERT**			Not tested
H7.d	7	5:1295135 G/A	ENSG00000164362	upstream gene variant	*TERT**			No tissue available
H7.e	7	5:1295135 G/A	ENSG00000164362	upstream gene variant	*TERT**			Yes

H8.a	8	5:150117795 C/T	ENSG00000113721	missense variant	*PDGFRB*	deleterious (0)	probably damaging (1)	Yes
H8.b	8	5:150117795 C/T	ENSG00000113721	missense variant	*PDGFRB*	deleterious (0)	probably damaging (1)	Yes
H8.c	8	5:150117795 C/T	ENSG00000113721	missense variant	*PDGFRB*	deleterious (0)	probably damaging (1)	Yes
H8.c	8	17:7675223 A/C	ENSG00000141510	missense variant	*TP53*	deleterious (0)	probably damaging (1)	Not validated
H8.a	8	17:7675223 A/C	ENSG00000141510	missense variant	*TP53*	deleterious (0)	probably damaging (1)	Yes
H8.b	8	17:7675223 A/C	ENSG00000141510	missense variant	*TP53*	deleterious (0)	probably damaging (1)	Yes
H8.a	8	5:1295113 G/A	ENSG00000164362	upstream gene variant	*TERT**			Not tested
H8.b	8	5:1295113 G/A	ENSG00000164362	upstream gene variant	*TERT**			Yes
H8.c	8	5:1295113 G/A	ENSG00000164362	upstream gene variant	*TERT**			Not tested
H9.a	9	5:1295113 G/A	ENSG00000164362	upstream gene variant	*TERT**			Not tested
H9.b	9	5:1295113 G/A	ENSG00000164362	upstream gene variant	*TERT**			Not tested
H9.c	9	5:1295113 G/A	ENSG00000164362	upstream gene variant	*TERT**			Not tested
H9.d	9	5:1295113 G/A	ENSG00000164362	upstream gene variant	*TERT**			Not tested
H9.e	9	5:1295113 G/A	ENSG00000164362	upstream gene variant	*TERT**			Not tested
H9.f	9	5:1295113 G/A	ENSG00000164362	upstream gene variant	*TERT**			Yes
H10.a	10	3:41224607 A/C	ENSG00000168036	missense variant	*CTNNB1*	deleterious (0)	probably damaging (0.959)	Yes
H10.b	10	3:41224607 A/C	ENSG00000168036	missense variant	*CTNNB1*	deleterious (0)	probably damaging (0.959)	Yes
H10.c	10	3:41224607 A/C	ENSG00000168036	missense variant	*CTNNB1*	deleterious (0)	probably damaging (0.959)	Yes
H10.d	10	3:41224607 A/C	ENSG00000168036	missense variant	*CTNNB1*	deleterious (0)	probably damaging (0.959)	Yes
H10.e	10	3:41224607 A/C	ENSG00000168036	missense variant	*CTNNB1*	deleterious (0)	probably damaging (0.959)	Yes
H10.a	10	17:7674885 C/A	ENSG00000141510	missense variant	*TP53*	deleterious (0)	probably damaging (0.998)	Yes
H10.a	10	17:7674888 T/G	ENSG00000141510	missense variant	*TP53*	deleterious (0)	probably damaging (1)	Yes
H10.b	10	17:7674885 C/A	ENSG00000141510	missense variant	*TP53*	deleterious (0)	probably damaging (0.998)	Yes
H10.b	10	17:7674888 T/G	ENSG00000141510	missense variant	*TP53*	deleterious (0)	probably damaging (1)	Yes
H10.c	10	17:7674885 C/A	ENSG00000141510	missense variant	*TP53*	deleterious (0)	probably damaging (0.998)	Yes
H10.c	10	17:7674888 T/G	ENSG00000141510	missense variant	*TP53*	deleterious (0)	probably damaging (1)	Yes
H10.d	10	17:7674885 C/A	ENSG00000141510	missense variant	*TP53*	deleterious (0)	probably damaging (0.998)	Yes
H10.d	10	17:7674888 T/G	ENSG00000141510	missense variant	*TP53*	deleterious (0)	probably damaging (1)	Yes
H10.e	10	17:7674885 C/A	ENSG00000141510	missense variant	*TP53*	deleterious (0)	probably damaging (0.998)	Yes
H10.e	10	17:7674888 T/G	ENSG00000141510	missense variant	*TP53*	deleterious (0)	probably damaging (1)	Yes
H10.a	10	5:1295113 G/A	ENSG00000164362	upstream gene variant	*TERT**			Not tested
H10.b	10	5:1295113 G/A	ENSG00000164362	upstream gene variant	*TERT**			Not tested
H10.c	10	5:1295113 G/A	ENSG00000164362	upstream gene variant	*TERT**			Not tested
H10.d	10	5:1295113 G/A	ENSG00000164362	upstream gene variant	*TERT**			Not tested
H10.e	10	5:1295113 G/A	ENSG00000164362	upstream gene variant	*TERT**			Not tested

*Due to low coverage, hot spot TERT mutations were called upon manual inspection of IGV

Supplemental Table 3. Selection of candidate expressed mutations to validate on DNA (Sanger Seq)

Sample code	Patient	Gene	Chr.	Mutation position (hg38)	Ref. allele	Alt. allele	Read depth (adj. tissue)	Read depth (tumor tissue)	Variant allele fraction (adj. tissue)	Variant allele fraction (tumor tissue)	Validated with Sanger DNA sequencing
H2.a	2	*ABCB11*	chr2	168970146	C	A	77	59	0	0.458	Yes
H2.d			chr2	168970146	C	A	77	44	0	0.432	Yes
H2.e			chr2	168970146	C	A	77	31	0	0.484	Yes
H2.b	2	*PCBP1*	chr2	70088071	G	T	205	138	0.004878	0.413	Yes
H2.c			chr2	70088071	G	T	205	102	0.004878	0.46	Yes
H2.d			chr2	70088071	G	T	205	123	0.004878	0.455	Yes
H3.a	3	*LEAP2*	chr5	132874044	G	C	125	107	0.008	0.523	No tissue available
H3.b			chr5	132874044	G	C	125	84	0.008	0.417	Yes
H3.a	3	*NEDD4L*	chr18	58367805	C	T	18	31	0	0.548	No tissue available
H3.b			chr18	58367805	C	T	18	43	0	0.535	Yes
H4.a	4	*SUZ12*	chr17	31994610	A	G	12	13	0	0.538	Yes
H4.e			chr17	31994610	A	G	12	13	0	0.462	Yes
H4.b	4	*EPHX1*	chr1	225845238	T	C	182	114	0.005495	0.439	Yes
H4.e			chr1	225845238	T	C	182	130	0.005495	0.554	Yes
H8.a	8	*INSIG1*	chr7	155302354	G	A	114	136	0.008772	0.426	Yes
H8.c			chr7	155302354	G	A	114	143	0.008772	0.406	Yes
H8.a	8	*SLC25A4*	chr4	185146940	T	A	50	48	0	0.438	Yes
H8.b			chr4	185146940	T	A	50	49	0	0.49	Yes
H8.c			chr4	185146940	T	A	50	48	0	0.479	Yes
H10.a	10	*SLC4A4*	chr4	71357019	G	T	10	29	0	0.655	Yes
H10.b			chr4	71357019	G	T	10	16	0	0.625	Yes
H10.c			chr4	71357019	G	T	10	15	0	0.533	Yes
H10.e			chr4	71357019	G	T	10	23	0	0.696	Yes
H10.a	10	*WDR6*	chr3	49015681	C	A	90	36	0	0.556	Yes
H10.c			chr3	49015681	C	A	90	58	0	0.569	Yes
H10.d			chr3	49015681	C	A	90	63	0	0.476	Yes
H10.e			chr3	49015681	C	A	90	41	0	0.488	Yes
H10.c	10	*AR*	chrX	67723710	A	G	35	19	0.029	1	Yes

Supplemental Table 4. HBV insertions in the multi-regional sampled HCC dataset

Sample code	Tissue	Partner1	Partner2	Score	Discordant reads	Split reads	NearGene1	NearGene2	Distance1	Distance2
N1.a	Adjacent	chrHBV:1802:+	chr3:96151968:-	4.2	0	206		AC108739.1	0	14845
N1.a	Adjacent	chr21:16288549:+	chrHBV:1826:-	1.822857	0	68	MIR99AHG		0	0
N1.b	Adjacent	chrHBV:1817:+	chr2:229018872:-	1.8	0	41		PID1	0	0
N1.c	Adjacent	chr2:236228953:+	chrHBV:1742:-	0.6	0	7	AC079135.1		0	0
N1.c	Adjacent	chrHBV:1787:+	chr7:136827588:-	0.649143	0	8		AC009264.1	0	0
N1.d	Adjacent	chr2:143204064:-	chrHBV:1828:+	2.05	0	43	ARHGAP15		0	0
N1.d	Adjacent	chr14:68720012:+	chrHBV:1826:-	0.25	0	14	RAD51B		0	0
H2.a	HCC	chr8:36867735:+	chrHBV:339:+	0.7	0	10	KCNU1		0	0
H2.a	HCC	chrHBV:1783:+	chr8:37017599:+	2.285714	0	40		AC090453.1	0	-29187
H2.b	HCC	chr8:37017599:-	chrHBV:1783:-	23.74514	0	373	AC090453.1		-29187	0
H2.b	HCC	chr8:36863519:+	chrHBV:343:+	0.4	0	6	KCNU1		0	0
H2.b	HCC	chrHBV:339:-	chr8:36863519:-	0.5	0	8		KCNU1	0	0
H2.b	HCC	chr8:36867735:+	chrHBV:339:+	2.5	0	33	KCNU1		0	0
H2.c	HCC	chr8:36867735:+	chrHBV:343:+	0.8	0	8	KCNU1		0	0
H2.c	HCC	chrHBV:339:-	chr8:36867735:-	0.8	0	16		KCNU1	0	0
H2.c	HCC	chrHBV:339:-	chr8:36863519:-	0.5	0	9		KCNU1	0	0
H2.c	HCC	chrHBV:1783:+	chr8:37017599:+	10.54286	0	147		AC090453.1	0	-29187
H2.d	HCC	chr8:37017599:-	chrHBV:1783:-	32.22743	0	437	AC090453.1		-29187	0
H2.d	HCC	chrHBV:1941:-	chr8:36870690:-	0.66	0	12		KCNU1	0	0
H2.d	HCC	chr8:36867735:+	chrHBV:343:+	1.9	0	26	KCNU1		0	0
H2.d	HCC	chrHBV:339:-	chr8:36863519:-	0.3	0	8		KCNU1	0	0
H2.d	HCC	chr8:36867735:+	chrHBV:339:+	1.1	0	24	KCNU1		0	0
N2.a	Adjacent	chrHBV:1814:+	chr4:44167599:+	0.25	0	12		KCTD8	0	6310
N2.a	Adjacent	chr2:215414958:+	chrHBV:458:-	0.279429	0	9	FN1		0	0
N2.a	Adjacent	chr2:231458389:+	chrHBV:458:-	2.4	0	41	NCL		0	0
N2.a	Adjacent	chrHBV:1805:+	chr10:23099289:-	0.326857	0	6		MSRB2	0	0
N2.b	Adjacent	chrHBV:1546:+	chr1:97283110:+	2.153143	0	66		DPYD	0	0
H3.b	HCC	chr6:24482294:+	chrHBV:1826:-	0.65	0	15	GPLD1		0	0
N3	Adjacent	chrHBV:1819:+	chr7:120346064:-	0.8	0	22		KCND2	0	0
H4.a	HCC	chrHBV:1913:-	chr3:10406683:-	0.35	0	11		ATP2B2	0	0
N4	Adjacent	chr4:85490163:-	chrHBV:1804:-	6.7	0	121	ARHGAP24		0	0
N4	Adjacent	chr9:88786306:+	chrHBV:1784:-	4.103429	0	66	MIR4289		-40401	0
N5	Adjacent	chrHBV:1755:+	chr1:244924554:+	4	0	136		RN7SKP55	0	19356
N5	Adjacent	chrHBV:458:+	chr8:100718280:-	9.829143	0	139		PABPC1	0	0
N5	Adjacent	chrHBV:1819:-	chr8:100720866:+	0.6	0	7		PABPC1	0	0
H10.a	HCC	chrHBV:458:+	chr1:30253343:+	0.15	0	7		RP3-357I16.1	0	-26728
H10.a	HCC	chr1:30233637:-	chrHBV:458:-	1.481143	0	16	RP3-357I16.1		-7022	0
H10.b	HCC	chr1:30233637:-	chrHBV:458:-	1.152	0	18	RP3-357I16.1		-7022	0
H10.c	HCC	chr6:42822018:-	chrHBV:458:-	1.457143	0	18	GLTSCR1L		0	0
H10.c	HCC	chr1:30233637:-	chrHBV:458:-	1.4	0	19	RP3-357I16.1		-7022	0
H10.c	HCC	chrHBV:1718:+	chr1:30233637:+	0.579429	0	7		RP3-357I16.1	0	-7022
H10.d	HCC	chr1:30233637:-	chrHBV:458:-	1.081143	0	22	RP3-357I16.1		-7022	0
H10.e	HCC	chrHBV:458:+	chr6:42810306:+	0.177143	0	6		GLTSCR1L	0	0
H10.e	HCC	chr6:42822018:-	chrHBV:458:-	1.8	0	24	GLTSCR1L		0	0
H10.e	HCC	chr1:30233637:-	chrHBV:458:-	0.290286	0	6	RP3-357I16.1		-7022	0
N10.b	Adjacent	chrHBV:1802:+	chr5:17918348:+	0.388571	0	6		RP11-454P21.1	0	0

Supplemental Table 5. List of primers used for Sanger sequencing

Gene	Ensembl ID	Chr.	Sequence (5' to 3')
TERT Prom	ENSG00000164362	5	CAGCGCTGCCTGAAACTC
TERT Prom	ENSG00000164362	5	GTCCTGCCCCTTCACCTT
CTNNB1	ENSG00000168036	3	GATTTGATGGAGTTGGACATGG
CTNNB1	ENSG00000168036	3	TGTTCTTGAGTGAAGGACTGAG
PDGFRB(I)	ENSG00000113721	22	GCCCGGAACAATATGCCAAGA
PDGFRB (I)	ENSG00000113721	22	TCGGTTTTCACACTGATTCCTG
PDGFRB(II)	ENSG00000113721	22	GGTCAGGCAGGGGGATGATA
PDGFRB (II)	ENSG00000113721	22	GGGATGGGCTCGGTTAGAAG
TP53 (I)	ENSG00000141510	5	TGTTCACTTGTGCCCTGACT
TP53 (I)	ENSG00000141510	5	AACCAGCCCTGTCGTCTCT
TP53 (II)	ENSG00000141510	6	TTACTTTGCACATCTCATGGGG
TP53 (II)	ENSG00000141510	6	GCTGCTCAGATAGCGATGGT
ATM	ENSG00000149311	11	TCCCCCTGTTATACCCAGTTG
ATM	ENSG00000149311	11	TCTGCTACCACTGCTTCAAAT
ARIDIA	ENSG00000117713	1	CCTCAACCAACTGCTCACG
ARIDIA	ENSG00000117713	1	AAACCCAACACAAAGGCTGAAG
JAK1	ENSG00000162434	1	CCAGCCTTGCATAACATACCG
JAK1	ENSG00000162434	1	GCAAGTACAGTCCAGGTGAGA
ABCB11	ENSG00000073734	2	GCAGCACAAGCATTTCCACAT
ABCB11	ENSG00000073734	2	TTTACACAGCCCAGTAAACCT
AR	ENSG00000169083	X	GGGAGGAAACAAAAGGCTGAAA
AR	ENSG00000169083	X	ACACAGATAGGAGCCACAATGG
EPHX1	ENSG00000143819	1	CTCCGTCGGCTCTTTCACTT
EPHX1	ENSG00000143819	1	ACTGAGTTCCAGCCGCTTTT
INSIG1	ENSG00000186480	7	CCCATGTTTTTAACCCTTGTGGT
INSIG1	ENSG00000186480	7	ACTACTTGGTTTTCCTTTGCGT
LEAP2	ENSG00000164406	5	TACAGCATCTGCGGAATGGA
LEAP2	ENSG00000164406	5	ATGGAAAGGCACCACTCTTTCA
NEDD4L	ENSG00000049759	18	AGGTACAGAATGCTCGCAGG
NEDD4L	ENSG00000049759	18	AGTGCCCAAAATAAGCTACCAGA
PCBP1	ENSG00000169564	2	GGATCAACATCTCGGAGGGG
PCBP1	ENSG00000169564	2	TGACACACTCGGTGACAGAC
SLC25A4	ENSG00000151729	4	TTTCCTCCAGCGTTACGGAG
SLC25A4	ENSG00000151729	4	GAAAGCGTGCATTAAGTGGTCT
SLC4A4	ENSG00000080493	4	CAATGCCCATATCTGTGTACCCT
SLC4A4	ENSG00000080493	4	ACAGGAGACGGTGTAAAGGTG
SUZ12	ENSG00000178691	17	CTGGTGTGCCTGGCCTATTTT
SUZ12	ENSG00000178691	17	GCCCTTTTTACCTACCTGCACA
WDR6	ENSG00000178252	3	CGACTCCCCATGACAAGACA
WDR6	ENSG00000178252	3	GGCATAGACAACCAGGACACT

Supplemental Table 6. List of genes included in the capture panel for targeted DNA

Gene	Ensembl ID	Chr.	Coordinates (hg38)	
			Gene start	Gene end
ACVR2A	ENSG00000121989	2	147844517	147930826
ALB	ENSG00000163631	4	73397114	73421412
APOB	ENSG00000084674	2	21001429	21044073
ARID1A	ENSG00000117713	1	26693236	26782104
ARID2	ENSG00000189079	12	45729665	45908040
ATM	ENSG00000149311	11	108222484	108369102
AXIN1	ENSG00000103126	16	287440	352673
BRAF	ENSG00000157764	7	140719327	140924764
CCND1	ENSG00000110092	11	69641087	69654474
CCNE1	ENSG00000105173	19	29811989	29824308
CDK4	ENSG00000135446	12	57747727	57756013
CDK6	ENSG00000105810	7	92604921	92836694
CDKN2A	ENSG00000147889	9	21967753	21995301
CDKN2B	ENSG00000147883	9	22002903	22009363
CTNNB1	ENSG00000168036	3	41194837	41260096
ERBB3	ENSG00000065361	12	56079857	56103505
EGFR	ENSG00000146648	7	55019021	55211628
FEZF2	ENSG00000153266	3	62369681	62374324
FGA	ENSG00000171560	4	154583126	154590766
FGF19	ENSG00000162344	11	69698232	69704642
FGF3	ENSG00000186895	11	69810224	69819024
FGF4	ENSG00000075388	11	69771016	69775403
FLT4	ENSG00000037280	5	180601506	180649624
FOXI3	ENSG00000214336	2	88446787	88452656
FZR1	ENSG00000105325	19	3506273	3538330
H1FNT	ENSG00000187166	12	48328980	48330279
HEY1	ENSG00000164683	8	79764010	79767863
HNF1A	ENSG00000135100	12	120978543	121002512
HRAS	ENSG00000174775	11	532242	537287
IDH1	ENSG00000138413	2	208236227	208266074
IL6ST	ENSG00000134352	5	55935095	55994993
IRF2	ENSG00000168310	4	184387713	184474580
JAK1	ENSG00000162434	1	64833229	64966504
JAK3	ENSG00000105639	19	17824780	17848071
KEAP1	ENSG00000079999	19	10486120	10503741
KMT2D	ENSG00000167548	12	49018975	49059774
KRAS	ENSG00000133703	12	25204789	25250936
MET	ENSG00000105976	7	116672390	116798386
MYC	ENSG00000136997	8	127735434	127741434
NCOR1	ENSG00000141027	17	16029157	16218185
NFE2L2	ENSG00000116044	2	177227595	177392697
NRAS	ENSG00000213281	1	114704469	114716894
NTRK3	ENSG00000140538	15	87859751	88256768
PDGFRB	ENSG00000113721	5	150113837	150155872

PIK3CA	ENSG00000121879	3	179148114	179240093
PSMB11	ENSG00000222028	14	23042167	23044276
PTEN	ENSG00000171862	10	87863113	87971930
PTPN3	ENSG00000070159	9	109375466	109498313
RB1	ENSG00000139687	13	48303751	48481986
RET	ENSG00000165731	10	43077027	43130351
RPS6KA3	ENSG00000177189	X	20149911	20267100
SMC3	ENSG00000108055	10	110567691	110604636
SRCAP	ENSG00000080603	16	30698209	30741409
TERT promoter	NA	5	1295151	1295347
TP53	ENSG00000141510	17	7661779	7687550
TSC1	ENSG00000165699	9	132891348	132944633
TSC2	ENSG00000103197	16	2047465	2088720
VEGFA	ENSG00000112715	6	43770184	43786487

Supplemental Table 7. Final terms in the Cox regression model

<table>
<tr><th>Model including ITH signature (principal components)</th><th>Coef</th><th>S.E.</th><th>Z</th><th>Pr(>|Z|)</th></tr>
<tr><td>Clustering Coefficient (mean)</td><td>-25.736</td><td>21.2162</td><td>-1.213</td><td>0.2251</td></tr>
<tr><td>Neighborhood Connectivity (sd)</td><td>-0.4791</td><td>0.1626</td><td>-2.9475</td><td>0.0032</td></tr>
<tr><td>Neighborhood Connectivity (mean)</td><td>0.4042</td><td>0.1655</td><td>2.4426</td><td>0.0146</td></tr>
<tr><td>Immune Reads/Clone</td><td>2.5172</td><td>0.8466</td><td>2.9732</td><td>0.0029</td></tr>
<tr><td>HLA-I B expression</td><td>0.0015</td><td>6.00E-04</td><td>2.7345</td><td>0.0062</td></tr>
<tr><td>ITH signature (PC4)</td><td>-0.065</td><td>0.0141</td><td>-4.6155</td><td><0.0001</td></tr>
<tr><td>ITH signature (PC2)</td><td>-0.0088</td><td>0.0054</td><td>-1.6513</td><td>0.0987</td></tr>
<tr><td>Tumor clones (DNA)</td><td>0.2872</td><td>0.0712</td><td>4.0354</td><td>1.00E-04</td></tr>
<tr><td>CD8A expression</td><td>-0.4155</td><td>0.1163</td><td>-3.5719</td><td>4.00E-04</td></tr>
<tr><td>ITH signature (PC1)</td><td>-0.0334</td><td>0.0071</td><td>-4.673</td><td><0.0001</td></tr>
<tr><td>DNA mutation count</td><td>0.217</td><td>0.1331</td><td>1.631</td><td>0.1029</td></tr>
<tr><td>CTLA4 expression</td><td>-0.1655</td><td>0.0971</td><td>-1.7038</td><td>0.0884</td></tr>
<tr><td>CD274 expression</td><td>-0.2083</td><td>0.1313</td><td>-1.5857</td><td>0.1128</td></tr>
</table>

<table>
<tr><th>Model including G3 signature</th><th>Coef</th><th>S.E.</th><th>Z</th><th>Pr(>|Z|)</th></tr>
<tr><td>G3 class</td><td>1.1829</td><td>0.2769</td><td>4.2716</td><td><0.0001</td></tr>
<tr><td>Tumor Clones (DNA)</td><td>0.2214</td><td>0.0661</td><td>3.3483</td><td>8.00E-04</td></tr>
<tr><td>Immune Reads/Clone</td><td>1.6453</td><td>0.7704</td><td>2.1357</td><td>0.0327</td></tr>
<tr><td>HLA-I A expression</td><td>0.0018</td><td>6.00E-04</td><td>3.0726</td><td>0.0021</td></tr>
<tr><td>CD8A expression</td><td>-0.2012</td><td>0.0817</td><td>-2.4639</td><td>0.0137</td></tr>
<tr><td>Neighborhood Connectivity (sd)</td><td>-0.3772</td><td>0.1447</td><td>-2.6078</td><td>0.0091</td></tr>
<tr><td>Neighborhood Connectivity (mean)</td><td>0.3119</td><td>0.1412</td><td>2.2096</td><td>0.0271</td></tr>
<tr><td>Clustering Coefficient (mean)</td><td>-22.6871</td><td>18.1191</td><td>-1.2521</td><td>0.2105</td></tr>
</table>
