# Supplementary material for: Intratumoral heterogeneity and clonal evolution in liver cancer
Source: Nat Commun. 2020 Jan 15;11:291. doi: 10.1038/s41467-019-14050-z (PMC6962317; doi:10.1038/s41467-019-14050-z)
Supplement: Supplementary file 2 — Description of Additional Supplementary Files [file 41467_2019_14050_MOESM2_ESM.pdf]

Description of Additional Supplementary Files

File Name: Supplementary Data 1
Description: Expressed somatic mutations in the multi-regional sampled HCC dataset

File Name: Supplementary Data 2
Description: HCC regional neo-epitopes and putative immunogenicity

File Name: Supplementary Data 3
Description: ITH signature

File Name: Supplementary Data 4
Description: Differentially expressed genes across single cell sequencing clusters

File Name: Supplementary Data 5
Description: Percentage of HCC cells with predicted transcription factors “on” as per SCENIC analysis
