# Supplementary material for: Intratumoral heterogeneity and clonal evolution in liver cancer
Source: Nat Commun. 2020 Jan 15;11:291. doi: 10.1038/s41467-019-14050-z (PMC6962317; doi:10.1038/s41467-019-14050-z)
Supplement: Supplementary file 8 — Reporting Summary [file 41467_2019_14050_MOESM8_ESM.pdf]

nature research

Corresponding author(s): Augusto Villanueva

Last updated by author(s): Dec 5, 2019

Reporting Summary

Nature Research wishes to improve the reproducibility of the work that we publish. This form provides structure for consistency and transparency in reporting. For further information on Nature Research policies, see Authors & Referees and the Editorial Policy Checklist.

Statistics

For all statistical analyses, confirm that the following items are present in the figure legend, table legend, main text, or Methods section.

n/a	Confirmed	
☐	☒	The exact sample size (*n*) for each experimental group/condition, given as a discrete number and unit of measurement
☐	☒	A statement on whether measurements were taken from distinct samples or whether the same sample was measured repeatedly
☐	☒	The statistical test(s) used AND whether they are one- or two-sided<br>*Only common tests should be described solely by name; describe more complex techniques in the Methods section.*
☐	☒	A description of all covariates tested
☐	☒	A description of any assumptions or corrections, such as tests of normality and adjustment for multiple comparisons
☐	☒	A full description of the statistical parameters including central tendency (e.g. means) or other basic estimates (e.g. regression coefficient) AND variation (e.g. standard deviation) or associated estimates of uncertainty (e.g. confidence intervals)
☐	☒	For null hypothesis testing, the test statistic (e.g. *F*, *t*, *r*) with confidence intervals, effect sizes, degrees of freedom and *P* value noted<br>*Give P values as exact values whenever suitable.*
☐	☒	For Bayesian analysis, information on the choice of priors and Markov chain Monte Carlo settings
☐	☒	For hierarchical and complex designs, identification of the appropriate level for tests and full reporting of outcomes
☐	☒	Estimates of effect sizes (e.g. Cohen's *d*, Pearson's *r*), indicating how they were calculated

Our web collection on statistics for biologists contains articles on many of the points above.

Software and code

Policy information about availability of computer code

Data collection

Mutation Annotation Files (MAF) and RNA-Seq FASTQs for the TCGA dataset (LIHC cohort) were downloaded from the National Cancer Institute's GDC Data Portal (https://portal.gdc.cancer.gov/) for HCC patients. Matched clinical data was downloaded from the cBioPortal (http://www.cbioportal.org/).

Data analysis

WES data processing was performed using a custom nextflow pipeline that is available on GitHub (https://github.com/losiclab/exoseq). Raw reads were trimmed and aligned to the hg38 reference genome using trim-galore53,54, bwa mem and samtools, respectively. Duplicate reads were then marked using picard MarkDuplicates, and bam quality scores were recalibrated for known technical bias using GATK4 base recalibration. Quality control metrics were compiled using fastqc2 for raw reads and picard CollectMultiMetrics for aligned reads. Somatic variants were called using Mutect2 in WES tumor-normal matched data, with the intervals parameter set to all coding regions, and with 1000 genomes as the germline resource. Variants were filtered for quality control using GATK FilterMutectCalls with default parameters. Only variants with a VAF > 5% were retained for further analysis. Variants were annotated with Annovar (version 2019 Apr09).
Copy number variation was studied at the level of allele-specific variation (ASCAT version 2.4) and at whole copy number variation (Circular Binary
Segmentation, CBS, relying exclusively on Log R Ratios of cases vs. controls). We used ASCAT to dissect the allele-specific copy number alterations, while simultaneously estimating and adjusting for both tumor ploidy and non-aberrant cell admixture. To analyze focal events of the copy number alteration (CNA) profiles, we used as input the average Log R Ratios per segment obtained from CBS.
RNA-seq:
Raw sequencing reads were mapped to the GRCh38 reference genome (USCS) using STAR (version 2.4.2g1). Aligned reads were mapped to
GRCh38 genetic features using featureCounts from the subRead package with default settings, with a median coverage of 30 million mapped reads per region.
Neo-antigen calling:
Raw sequencing reads were re-mapped to all known HLA-I alleles using a 4-step approach. First, a low-stringency mapping was performed using razers3 to identify HLA-matching reads. If there were more than 10,000 such HLA reads, they were next randomly down

sampled to produce a more manageable, smaller output file (with a maximum of 10,000 reads). For step three, Optitype (version 1.0) was used on this
low-stringency, potentially down sampled HLA-specific, razers3 output to consensus call the HLA alleles. Finally, the overlap of reads mapping to distinct alleles was quantified by assigning fractions of read support to each allele using a custom script that operates only on the high-stringency mapping output of Optitype.
Mapped RNA-seq reads were subject to splitting, trimming, local indel realignment, and base-score recalibration pre-processing with the IndelRealigner and TableRecalibration tools from GATK under the GATK Best Practices for RNA-seq paradigm. Mutect (version 2.0) was then used to
compute the regional somatic mutation burden in the following fashion. For each patient, all reads from adjacent non-tumoral regions were combined to form an effective normal against which tumor regions were tested for somatic mutations. Somatic calls from mutect with fewer than 10 supporting variant reads were not considered. To predict neo-antigen and associated epitope burden, we used Topiary (Rubinsteyn and Nathanson, https://github.com/hammerlab/topiary) to call mutation-derived cancer T-cell epitopes from somatic variants, tumor RNA expression data, and patient class I HLA type. the peptide up to the first stop codon.
TCR calling:
DNA: T cell receptor beta clonality and richness values were obtained through the ImmunoSeq Analyzer software.
RNA: Mapped RNA sequencing reads were used to allelotype (MHC class-I loci) each patient, estimate the putative TIL burden per patient by profiling TCR and BCR sequences with MiXCR, and normalizing by patient library size.
HBV calling:
Raw RNA sequencing reads that did not map to the GRCh38 reference genome was assembled into contigs using Trinity (using –no_run_chrysalis –no_run_butterfly flags, which effectively only invokes Inchworm) to perform greedy kmer-21 contig assembly. Contigs with a sufficiently high entropy (to exclude homopolymer sequences), at least 100 bp long and supported by at least 20 reads were retained for further analysis. Contigs were BLASTed (BLAST version 2.2.26+) to HBV sequence and all contigs with bitscore >= 100 were retained.
We then took that specific HBV genome in its entirety and first computed the longest open reading frames using TransDecoder.LongOrfs and then predicted likely coding regions (CDS) using TransDecoder.Predict at default settings. We examined the chimeric alignments from the initial regional STAR alignments and post-filtered them with an emphasis on precision using STARChip (version 1.1).
For regional expression variance analysis, in order to facilitate gene ranking, stable effect size estimation, and variance sharing across genes among samples we used DESeq2 to model the dependence of the dispersion of the count data on the average expression strength over all of the samples in the comparison. Gene set enrichment analysis (GSEA) was used to determine if a gene list composed of expressed CTAs shows cumulative changes in expression across our ITH dataset.
TCGA RNA-Seq data was aligned to Hg38 with STAR (v2.5.1b) in two-pass mode. Gene counts for Gencode v23 (www.gencodegenes.org) gene annotations were generated using featureCounts. Read counts underwent TMM normalization and logCPM transformation using voom.
Analysis of the single cell RNA-seq data was done with the package Seurat (version 2.1). Non-linear dimensionality reduction techniques like t-SNE and
topological data analysis were done using TDA and the Ayasdi Platform. The SCENIC workflow was used to determine transcription factor co-expression modules.
For our survival analysis, we used the Nearest Template Prediction method to determine which patients in the TCGA-HCC dataset had a significant enrichment of the ITH signatures. We built parametric Cox proportional hazard models via the rms R package by optimizing stepwise AIC across the model space of correlates.

For manuscripts utilizing custom algorithms or software that are central to the research but not yet described in published literature, software must be made available to editors/reviewers. We strongly encourage code deposition in a community repository (e.g. GitHub). See the Nature Research guidelines for submitting code & software for further information.

Data

Policy information about availability of data

All manuscripts must include a data availability statement. This statement should provide the following information, where applicable:

- Accession codes, unique identifiers, or web links for publicly available datasets
- A list of figures that have associated raw data
- A description of any restrictions on data availability

We have included a Data Availability Statement where we include all the information and details on how to access the raw data used in this study.

Field-specific reporting

Please select the one below that is the best fit for your research. If you are not sure, read the appropriate sections before making your selection.

☒ Life sciences ☐ Behavioural & social sciences ☐ Ecological, evolutionary & environmental sciences

For a reference copy of the document with all sections, see nature.com/documents/nr-reporting-summary-flat.pdf

Life sciences study design

All studies must disclose on these points even when the disclosure is negative.

Sample size

This is a pilot and exploratory study so no specific power calculation was conducted. The number of samples analyzed was within the range of similar studies evaluating ITH in human cancer (Gerlinger et al., NEJM 2012; Lin et al., Cancer Research 2017). The number of samples included was based on the number of samples available for analysis when the study was designed. The study is descriptive by nature, so we did not included sample size calculation as we did not have a pre-specified priors in terms of the predicted difference in magnitude for any of the molecular parameters we studied.

Data exclusions	All samples were included in the final analysis. The only exclusions for certain data points (e.g., DNA copy number changes) were due to the lack of enough tissue available for that particular sample. These exclusion criteria were pre-established as no analysis can be conducted if enough tissue is not available.
Replication	The study included multiregional sampling of 14 single-nodule hepatocellular carcinoma specimens. Each region sampled from the same nodule can be interpreted as a biological replicate of the tumor analyzed. In addition, we conducted a technical replicate of region A from patient 2 for the RNA-seq analysis. All attempts for replication were successful.
Randomization	The study is a descriptive analysis of 14 hepatocellular carcinoma resection specimens. Samples were randomly assigned to different lanes (RNA-seq and DNA targeted sequencing) and hybridization plates (SNP array). No randomization was applied, except when conducting the molecular analyses as decribed below.<br><br>For the expression analysis we included other covariate information such as RNA extraction, RIN numbers, sequencing batch etc as separate terms in the linear model in order to regress out expression variation associated with random, technical effects. Similarly in learning the ITH driven TCGA survival signatures we iteratively used nested cross-validation leveraging elastic net feature selection across randomized groups of regional samples and then iteratively quantified predictive error on randomized groups of TCGA patients.
Blinding	The ISMMS Tissue Biorepository provided de-identified samples.

Reporting for specific materials, systems and methods

We require information from authors about some types of materials, experimental systems and methods used in many studies. Here, indicate whether each material, system or method listed is relevant to your study. If you are not sure if a list item applies to your research, read the appropriate section before selecting a response.

Materials & experimental systems

n/a	Involved in the study	
☐	☒	Antibodies
☒	☐	Eukaryotic cell lines
☒	☐	Palaeontology
☒	☐	Animals and other organisms
☐	☒	Human research participants
☒	☐	Clinical data

Methods

n/a	Involved in the study	
☒	☐	ChIP-seq
☒	☐	Flow cytometry
☒	☐	MRI-based neuroimaging

Antibodies

Antibodies used	AlexaFluor® 488 (Invitrogen A21121 and A21212), AlexaFluor® 546 (Invitrogen A11030) and AlexaFluor® 594 (Invitrogen A11037), CD-3 (DAKO A0452), CD-20 (DAKO M0755), granulysin (Santa Cruz sc-271119), and PNAD (BD Biosciences 553863). Detaisl on dilustions and protocol are included in the methods section of the paper.
Validation	DAKO A0452 - representative citation for this antibody is Zhao et al., cancer mediates effector T cell dysfunction by targeting microRNAs and EZH2 via glycolysis restriction, Nature Immunology, 2016.<br>DAKO M0755 – representative citation for this antibody is Somasundaram et al., tumor associated B-cells induce tumor heterogeneity and therapy resistance, Nature Communications, 2017.<br>sc-271119 – representative citation for this antibody is Roncati et al., prognostic factors for breast cancer: an immunomorphological update, Pathology Oncology Research, 2016.<br>BD Biosciences 553863 - representative citation for this antibody is Kivisakk et al., human cerebrospinal fluid central memry CD4 + T cells: evidence for trafficking through choroid plexus and meninges via P-selectin, PNAS, 2003.

Human research participants

Policy information about studies involving human research participants

Population characteristics	All patients except P09 had single-nodule early stage HCC (Barcelona Clinic Liver Cancer stage A) and were treated with surgical resection without any prior therapy. Most patients were male (64%, 9/14), with a median age of 63 years.
Recruitment	All patients with liver cancer who undergo a surgical resection at Mount Sinai Hospital are offered to participate in the Tissue Biorepository. There are no major exclusion criteria other than the patient not consenting to provide their left-over surgical material for research purposes. As only patients at early stages are treated with resection, our results are restricted to these patients and should not be extrapolated to other disease stages (this is detailed in the Methods section). Besides this, there are no other potential bias for recruitment in this dataset.
Ethics oversight	Study was approved by the Mount Sinai IRB (IRB# HS-14-01011) and samples were provided by the ISMMS Tissue Biorepository (IRB# HS-10-00135) Biorepository.

Note that full information on the approval of the study protocol must also be provided in the manuscript.
